# Antibody-opsonized extracellular traps modulate macrophage responses and attenuate their T cell-associated inflammatory and costimulatory characteristics

**DOI:** 10.3389/fimmu.2026.1867666

**Published:** 2026-09-29

**Authors:** Annemarie Kip, Eline Zwiers, Kelsy Waaijenberg, Wynand Alkema, Eric Meldrum, Maarten van der Linden

**Affiliations:** 1 Citryll B.V., Oss, Netherlands; 2 Tenwise B.V., Leiden, Netherlands

**Keywords:** anti-citrullinated histone H2A/H4 antibody, CD80/CD86, CXCL9, extracellular traps, IL-10, immune regulation, macrophages, phagocytosis

## Abstract

Macrophages and neutrophils coordinate inflammatory and reparative immunity, and dysregulated interactions between these cells contribute to the pathogenesis of immune-mediated inflammatory diseases. While extracellular traps (ETs), formed by neutrophils and other leukocytes, are enriched in citrullinated histones and exert significant pro-inflammatory effects, it remains unclear how distinct macrophage subsets interpret and resolve ET-derived signals. Here we show that macrophage subsets consistent with pro-inflammatory (GM-CSF + LPS + IFN-γ differentiated) or inflammation-resolving (M-CSF + IL-4 differentiated) characteristics, differentially engage with neutrophil-derived ETs and that antibody binding to ETs reshapes these interactions. ETs induce the release of pro-inflammatory cytokines and chemokines in both macrophage subtypes. While inflammation-resolving macrophages readily phagocytose ETs, pro-inflammatory macrophages exhibit limited uptake. Fc domain-dependent ET phagocytosis by both macrophage subsets is significantly enhanced when ETs are opsonized with an anti-citrullinated histone H2A and H4 monoclonal antibody. Opsonized ETs upregulate macrophage expression of genes encoding negative regulators of Mitogen-Activated Protein Kinase (MAPK) signaling and reduce expression of genes encoding pro-inflammatory receptors. Exposure to antibody-opsonized ETs following macrophage activation with LPS and IFN-γ attenuates the T cell-associated features of pro-inflammatory macrophages with reduced surface expression of CD80 and CD86 and decreased secretion of IL-12 and CXCL9. In inflammation-resolving macrophages antibody-opsonized ETs attenuate MAPK-dependent Toll-like receptor responses and inhibit secretion of CXCL9 and CXCL10 while enhancing IL-10 secretion. Together, these findings describe the pro-inflammatory impact of neutrophil-derived ET exposure on macrophages and delineate a previously unrecognized regulatory axis by which antibodies targeting ET components modulate macrophage responses across distinct subsets to attenuate their T cell-associated inflammatory and costimulatory responses, indicating relevance for therapeutic strategies aimed at modulating ET-driven inflammation.

## 1 Introduction

Macrophages are innate immune cells that maintain immune surveillance and tissue homeostasis through their clearance of apoptotic cells, cellular debris, and pathogens. They also secrete tissue repair factors to coordinate inflammation and reparative responses (1, 2). Macrophages exhibit phenotypic plasticity, adopting tissue-specific and context-dependent states across a spectrum of pro-inflammatory and inflammation-resolving characteristics, each recognized by distinct transcriptional signatures, metabolic profiles, and effector functions (3). This functional diversity enables macrophages to adapt to dynamic microenvironmental cues and to modulate immune responses appropriately.

Dysregulated macrophage activity contributes to the pathogenesis of immune-mediated inflammatory diseases (IMIDs). In rheumatoid arthritis (RA), macrophage populations shift towards pathogenic states and accumulate in inflamed synovial tissue where they secrete pro-inflammatory cytokines such as tumor necrosis factor alpha (TNFα), interleukin (IL)-1β, and IL-6 (4, 5). In addition to cytokine production, RA macrophages interact with neutrophils, fibroblasts, and lymphocytes to amplify synovitis and joint destruction (6).

Neutrophils are the first line of defense against invading pathogens and, like macrophages, disturbance in neutrophil activity is associated with the pathogenesis of IMIDs such as RA and hidradenitis suppurativa (HS) (7–9). Neutrophils infiltrating inflamed joints and skin lesions undergo a form of cell death characterized by cell rupture and the accumulation of chromatin in the extracellular environment. This chromatin is decorated with neutrophil bactericidal granule components, forming structures known as extracellular traps (ETs) (10, 11). ET formation requires chromatin decondensation (12), a process dependent on peptidylarginine deiminase 4 (PAD4)-mediated histone citrullination. ETs and their citrullinated components are pro-inflammatory, cytotoxic and immunogenic, promoting tissue injury and autoimmunity (13–15).

In addition to locally secreting deoxyribonucleases (DNases), macrophages contribute to the resolution of ET-driven inflammation by phagocytosing ETs (16, 17). The immunological consequences of ET uptake by macrophages are incompletely understood with some studies showing that ETs activate macrophage inflammatory pathways and enhance cytokine secretion (17–20) and others showing ET phagocytosis to be immunologically silent (16). Furthermore, how phagocytic efficiency and downstream consequences of ET clearance may differ across distinct macrophage subsets has not been addressed.

Antibodies targeting ETs or ET components, including high mobility group box 1, histones and citrullinated histones, have recently emerged as potential therapeutic strategies for IMIDs (21–28). Such antibodies can bind ETs, and in so doing may modulate macrophage responses and reshape inflammatory networks within inflamed tissues.

In this study, we investigate the response of distinct macrophage subsets, consistent with pro-inflammatory (Granulocyte-macrophage colony-stimulating factor (GM-CSF) + Lipopolysaccharide (LPS) + Interferon (IFN)-γ differentiated) or inflammation-resolving (Macrophage colony-stimulating factor (M-CSF) + IL-4 differentiated) characteristics. We examine how neutrophil-derived ETs influence macrophage subset-specific inflammatory responses and demonstrate that antibody-opsonization of ETs with an anti-citrullinated histone H2A and H4 (anti-citH2A/H4) monoclonal antibody significantly changes macrophage responses to engagement with ETs shifting the outcome, across distinct macrophage subsets, toward reduced T cell-associated inflammatory and costimulatory activity.

## 2 Methods

### 2.1 Primary monocyte isolation

Peripheral venous blood from healthy, de-identified donors was obtained from the Sanquin blood bank (The Netherlands) with written informed consent. Buffy coats were diluted 1:1 in DPBS containing 2 mM EDTA, and peripheral blood mononuclear cells were isolated by Ficoll-Paque Plus density-gradient centrifugation (25 min, 800 x g), followed by one wash in DPBS containing 2 mM EDTA (centrifugation for 10 min, 300 x g) and two washes in DPBS containing 2 mM EDTA and 0.5% BSA (centrifugation for 10 min, 200 x g). Monocytes were purified using CD14 MicroBeads (Miltenyi, 130-097-052) according to the manufacturer’s instructions and resuspended in IMDM (Thermo Fisher Scientific, 12440061) supplemented with 1% penicillin-streptomycin, 1% FBS (Serena, S-FBS-SA-015), and 1% L-glutamine (monocyte buffer). Live cell counts were obtained using trypan blue with a Cellometer Auto T4 Bright Field Cell Counter, and monocyte purity was confirmed by flow cytometry as described below.

### 2.2 Flow cytometry

Monocyte purity was assessed by flow cytometry. Cells were resuspended in cold DPBS containing 1% BSA and 0.1% NaN_3_ (FACS buffer) and pre-incubated with 50x diluted Human TruStain FcX (BioLegend, 422302) for 15 min at 4 °C. Samples were then diluted 1:1 in FACS buffer containing 0.5 µg/mL APC-H7-anti-CD14 (BD, 560180), 0.09 µg/mL APC-anti-CD45 (BioLegend, 304037), 0.0125 µg/mL PE–anti-CD41 (BD, 303706), and Fixable Viability Dye eFluor 506 (1:1000; Invitrogen, 65-0866-14). Cells were stained for 30 min at 4 °C and washed three times with FACS buffer (centrifugation for 3 min, 400 x g). Data were acquired on a BD FACSCanto™ II or FACSymphony™ A3 using FACSDiva (v9.3.1) and analyzed with FlowJo (v10.10.0). The gating strategy for the monocyte purity check is demonstrated in **Supplementary Figure 1**.

To assess FcγR surface expression, cells were stained as described above using 10 µg/mL anti-FcγRI blocking antibody (Biolegend, 305047, clone 10.1), anti-FcγRII blocking antibody (StemCell Technologies, 60012, clone IV.3), or anti-FcγRIII blocking antibody (Invitrogen, 16-0166-85, clone 3G8). Mouse anti-hen egg lysozyme IgG1κ or IgG2b antibodies (CrownVivo, C0005 and C0008, respectively) were used as isotype controls. Following incubation and washing, cells were incubated with 0.5 µg/mL of Alexa Fluor Plus 488 conjugated goat anti-mouse IgG antibody (Invitrogen, A32723) for 30 min at 4 °C. Cells were subsequently washed prior to analysis.

### 2.3 Production of neutrophil-derived ETs

Peripheral blood from healthy volunteers was collected in VACUETTE^®^ lithium-heparin tubes, neutrophils were isolated by Ficoll-Paque Plus density-gradient centrifugation, and neutrophil purity was assessed as described previously (28). Neutrophils were resuspended in RPMI 1640 (Gibco, 61870-010) supplemented with 2% FBS, 1% penicillin-streptomycin, 10 mM HEPES, and 1 mM CaCl_2_ (assay buffer), plated in 6-well plates (3 x 10^6^ cells per well), and stimulated with 6.25 - 12.5 µM A23187 for 3 h at 37 °C and 5% CO_2_ to induce ET formation. Wells were washed twice with DPBS, and ETs were digested with 15 U/mL micrococcal S7 nuclease (Thermo Fisher Scientific, EN0181) in assay buffer for 15 min at 37 °C. For the experiments involving macrophages exposure to soluble ETs, ETs were generated using 0.8 U/mL S7 nuclease in IMDM with 1% penicillin-streptomycin and 5% FBS for 5 min at 37 °C. Digestion was terminated by adding EDTA to a final concentration of 10 mM. To remove EDTA, ET preparations were subjected to buffer exchange using Amicon Ultra centrifugal filter units (Millipore, UFC801008D). ET preparations were 10x diluted in DPBS and centrifuged at 4, 000 x g for 15 min at room temperature (RT). Following centrifugation, DPBS was added as needed to restore the samples to their original volume. This buffer exchange procedure was repeated for a total of three cycles. An appropriate equivalent treatment negative control underwent the same processing steps as the ET preparation but did not contain ETs.

ET preparations were characterized by assessing DNA fragment size and citrullination levels as previously described (28), DNA-to-protein ratio using a NanoDrop spectrophotometer, endotoxin content using a recombinant factor C assay (Lonza, 50-658U) according to the manufacturer’s protocol, and the presence of MPO-DNA complexes, a well-established marker of neutrophil-derived ETs. The results of this characterization are presented in **Supplementary Figure 2**.

### 2.4 MPO-DNA ELISA

High-binding 96-well plates were coated overnight at 4 °C with 0.5 µg/mL mouse monoclonal anti-MPO antibody (Abcam, ab25989) diluted in PBS. Plates were washed three times with PBS containing 0.05% Tween-20 and blocked with 1% BSA for 2 h at RT with shaking (450 rpm). ET samples and buffer control, diluted in 1% BSA, were added to the wells and incubated for 2 h at RT with shaking. Following washing, in-house HRP-conjugated mouse monoclonal anti-DNA antibody (Abcam, ab27156) was added at 0.1 µg/mL and incubated for 1 h at RT with shaking. Plates were washed and developed with TMB for 10 min at RT in the dark. The reaction was stopped with 2 M H_2_SO_4_ and absorbance was measured at 450 nm with reference correction at 620 nm using a Tecan Infinite F50 plate reader.

### 2.5 Macrophage differentiation

Monocytes were plated according to downstream applications. For the experiments involving macrophages exposure to soluble ETs, 3.5 x 10^4^ monocytes per well were seeded in 96-well imaging plates in monocyte buffer, followed by centrifugation (5 min, 300 x g). After 1 h at 37 °C and 5% CO_2_, medium was replaced with IMDM containing 1% penicillin-streptomycin, 5% FBS, and either 50 ng/mL GM-CSF (Immunotools, 11343127; macrophages with pro-inflammatory characteristics) or 50 ng/mL macrophage colony-stimulating factor (M-CSF, Immunotools, 11343115; macrophages with inflammation-resolving characteristics). Cells were cultured for 3 days, with half-medium replacement on day 3. On day 6, macrophages were polarized by replacing medium with IMDM containing 1% penicillin–streptomycin, 5% FBS, and either, 100 ng/mL LPS O111:B4 and 25 ng/mL IFN-γ (consistent with a pro-inflammatory macrophage phenotype) or, 25 ng/mL IL-4 (consistent with an inflammation-resolving macrophage phenotype) for 24 h. Macrophage polarization was assessed by CD80 and CD163 surface expression (immunofluorescence microscopy) and by LPS-induced IL-6 and IL-10 detection in the supernatant, as described elsewhere in this study.

For the experiments involving macrophages exposure to immobilized ETs, monocytes were plated at 4.5 x 10^6^ cells per well in 6-well plates in monocyte buffer. After 1 h, medium was replaced with IMDM containing 1% penicillin-streptomycin, 5% FBS, and GM-CSF or M-CSF. On day 3, all medium was refreshed. The removed medium was centrifuged (5 min, 300 x g) to recover suspended cells, which were returned to the culture. On day 6, macrophages were polarized as described above.

### 2.6 Antibody production and F(ab’)2 fragment generation

Human IgG1*κ* anti-citrullinated histone H2A and H4 (anti-citH2A/H4) monoclonal antibody laboratory-scale batch, in previous publications referred to as CIT-013 (29) or development candidate therapeutic anti-citrullinated protein antibody (dc-tACPA) (27), has been produced as described elsewhere (28). The endotoxin level of anti-citH2A/H4 antibody was 0.0012 EU/mg protein. Anti-citH2A/H4 F(ab’)2 fragments were generated using the PierceTM F(ab’)2 preparation kit according to the manufacturer’s protocol. F(ab’)2 fragments were isolated using Praesto® Jetted A50 ProtA agarose (Purolite) affinity chromatography, and buffer exchange to PBS was performed. Quality of F(ab’)2 fragments was determined using SDS-PAGE and size-exclusion-high-performance liquid chromatography. The endotoxin level of F(ab’)2 fragment was 0.47 EU/mg protein.

### 2.7 Macrophage exposure to soluble ETs

To assess macrophage responses to soluble ETs, 500–2000 ng/mL ETs were resuspended in IMDM containing 1% penicillin-streptomycin and 5% FBS and added to macrophages. Plates were centrifuged (3 min, 200 x g) to facilitate ET-macrophage interaction, followed by incubation at 37 °C and 5% CO_2_ for 24 h. Macrophage cell viability was determined by CellTiter-Blue cell viability assay (Promega, G8080) according to the manufacturer’s protocol. CD80 and CD86 cell surface expression was determined by immunofluorescence microscopy, and cytokine release was assessed in the supernatant, as described elsewhere in this study.

### 2.8 ET phagocytosis

The ET phagocytosis assay used in this study is based on previously described methodology with minor modifications (28). To assess ET phagocytosis, 500 ng/mL ETs were preincubated with indicated concentrations of anti-citH2A/H4 monoclonal antibody or its corresponding F(ab’)2 fragments together with 1 µM Sytox Green. Macrophages were pretreated with 10 µM chloroquine in IMDM with 1% penicillin-streptomycin and 5% FBS for 15 min. ETs were added to the macrophages followed by centrifugation and incubation for 1 h at 37 °C and 5% CO_2_. Subsequently, macrophages were washed twice with DPBS and fixed in 2% PFA for 10 min at RT, followed by two additional DPBS washes. Macrophages were stained with CellMask Deep Red (1:50, 000; 10 min) and Hoechst 33342 (5 µM; 5 min) at 37 °C, then washed twice in DPBS. Imaging was performed on a CellInsight CX5 High-Content Screening platform, capturing nine fields per well or at least 600 cells. ET phagocytosis was analyzed with the Thermo Scientific HCS Studio 3 Cell Analysis Software using the colocalization measurement tool, identifying green intracellular puncta within the macrophage plasma membranes but outside the nuclei. The phagocytic index was calculated as the percentage of phagocytic macrophages multiplied by the number of positive spots per macrophage.

### 2.9 Macrophage exposure to immobilized ETs

To assess macrophage responses to immobilized ETs, allowing the selective analysis of Fc receptor (FcR)-driven responses independent of transcriptional changes associated with interaction with or internalization of soluble ETs, we established a standardized assay. ETs (125 ng/mL in DPBS) were immobilized onto 96-well plates overnight at 4 °C. Plates were washed two times with DPBS containing 0.05% Tween-20 and 1 mM EDTA (PBS+ buffer) and blocked for 1.5 h at RT with PBS+ buffer containing 2% BSA (blocking buffer). Indicated concentrations of anti-citH2A/H4 monoclonal antibody or its corresponding F(ab’)2 fragments, diluted in blocking buffer, were added and incubated for 1 h at RT with shaking (450 rpm). Plates were then washed three times with PBS+ buffer and three times with DPBS, and wells were maintained in DPBS until use.

Antibody or F(ab’)2 fragment binding to immobilized ETs was verified using HRP-conjugated goat anti-human IgG (Fab’)2 (Abcam, ab87422). After 1 h incubation at RT with shaking, wells were washed three times with PBS+ buffer, developed with TMB for 10 min at RT in the dark, and stopped with 2 M H_2_SO_4_. Absorbance was recorded on a Tecan Infinite F50 plate reader.

To harvest macrophages, culture supernatants were collected followed by two washes with cold DPBS. Cells were detached with TrypLE Express for 20–25 min at 37 °C, 5% CO_2_, and the enzyme was neutralized with cold IMDM containing 1% penicillin–streptomycin and 5% FBS. Supernatants and two vigorous cold DPBS washes were pooled on ice. Cells were pelleted (300 x g, 5 min, 4 °C) and resuspended in cold IMDM containing 1% penicillin–streptomycin and 5% FBS. Macrophages were counted (Cellometer Auto T4) and seeded onto immobilized ETs at 3.5 x 10^4^ cells per well. Plates were centrifuged (1 min, 100 x g) to promote ET-macrophage interaction before incubation at 37 °C and 5% CO_2_ for 6 h (RNA sequencing) or 24 h (CD80 and CD86 surface expression and analysis of cytokine secretion).

For experiments assessing the immunomodulatory effect of antibody-opsonized ETs following TLR stimulation, macrophages were incubated for 1 h with immobilized ETs and stimulated for 24 h with 10 ng/mL LPS O111:B4, 1 µg/mL Pam3CSK4, 10 µg/mL Poly(I:C), 1 µg/mL R848, 0.2 µg/mL serum amyloid a (SAA) or a combination of 100 ng/mL LPS O111:B4 and 25 ng/mL IFN-γ. When indicated, macrophages were preincubated for 15 min at RT with 10 µg/mL anti-FcγRI blocking antibody, anti-FcγRII blocking antibody, or anti-FcγRIII blocking antibody before macrophages were seeded onto immobilized ETs. Mouse anti-hen egg lysozyme IgG1κ or IgG2b antibodies were used as negative control. In a separate experiment, macrophages were pre-incubated with the MAPK2 inhibitor Zunsemetinib (MedChemExpress, CDD-450) for 1 h at 37 °C and 5% CO_2_ before stimulation with LPS.

### 2.10 RNA sequencing

After 6 h incubation, supernatant was removed and macrophages were collected in MagNA Pure External Lysis buffer and stored at -80 °C until processing. Low-input RNA sequencing was performed by GenomeScan (Leiden, The Netherlands) using Illumina NovaSeq X Plus with a reading depth of 5M reads, paired end (PE 150). GenomeScan provided raw Ensemble gene-level count matrices. No additional preprocessing or filtering was applied prior to analysis.

Gene expression analysis was performed in R (v4.3.3). Differential expression was assessed using the R package DESeq2 (v1.42.1) (30). Genes with very low counts were removed using DESeq2’s independent filtering, and library sizes were normalized using the median-of-ratios method. The design formula accounted for donor variability and included macrophage subtype, treatment, and their interaction: Design = ~ donor + macrophage subtype * treatment.

This framework enabled estimation of treatment effects within each macrophage subtype and the interaction term. Wald test contrasts were extracted, and p-values were adjusted using the Benjamini–Hochberg procedure. Genes were considered significantly regulated at adjusted p-value < 0.05 and |Log2 fold change| > 1.

Normalized counts were used for Principal component analysis (PCA) to assess sample clustering and potential batch effects. Differential expression patterns, heatmaps, and enrichment visualizations were generated in R using the R package ggplot2 (v3.5.0) (31).

Gene set enrichment analysis was performed with fgsea (v1.28.0) (32). The p-values were corrected for multiple testing using the Benjamini–Hochberg method.

### 2.11 Immunofluorescence microscopy analysis of protein surface expression

After 24 h incubation, macrophages were washed twice with DPBS and fixed in DPBS containing 2% PFA for 10 min at RT. Cells were washed three times with DPBS and blocked for 1 h at RT with 5% normal goat serum (NGS) in DPBS. Mouse anti-human CD80 antibody (2.5 µg/mL, BD, 557223), mouse anti-human CD86 antibody (2.5 µg/mL, BioLegend, 305402), and mouse anti-human CD163 antibody (1:100 diluted, Thermo Fisher Scientific, eBioGHI/61) were diluted in 2% NGS in DPBS and incubated overnight at 4 °C. Mouse anti-hen egg lysosome IgG1*κ* (CrownVivo, C0005) and mouse IgG2B isotype control (R&D Systems, MAB004) were used as negative control. After three washes with DPBS, Alexa Fluor Plus 488-conjugated goat anti-mouse IgG (10 µg/mL, Invitrogen, A-11001) diluted in 2% NGS in DPBS was added for 1 h at RT. Cells were washed again and stained with 5 µM Hoechst 33342 for 5 min in the dark, washed, and maintained in DPBS for imaging.

Imaging was performed on a CellInsight CX5 High-Content Screening platform, acquiring 9 fields per well or ≥600 cells per well. Staining intensity was quantified with the Thermo Scientific HCS Studio 3 Cell Analysis Software using the compartmental analysis tool. Signal remaining after background subtraction and exclusion of nuclear regions was considered positive. Total fluorescence intensity was normalized to the number of analyzed cells.

### 2.12 Cytokine and chemokine measurements

After 24 h incubation, macrophage supernatants were collected and the level of cytokines and chemokines were analyzed. Chemokine (C-X-C motif) ligand 9 (CXCL9) concentrations were quantified using the Human MIG/CXCL9 ELISA Kit (Invitrogen, EHCXCL9), and CXCL10 using the Human CXCL10/IP-10 Quantikine ELISA Kit (R&D Systems, DIP100). IL-8, TNFα, IL-6, IL-10, C-C motif ligand (CCL) 2, CCL3, IFN-γ, IL-1β, IL-4, IL-12p70, and IL-1Ra were measured with built-in triplicates using custom multiplex panels on the Ella Automated Immunoassay System (BioTechne). In some experiments, IL-6 and IL-10 were quantified with Human IL-6 and IL-10 Uncoated ELISA Kits (Invitrogen, 88-7066–88 and 88-7106-88). All assays were performed according to the manufacturers’ instructions. Absorbance was recorded on a Tecan Infinite F50 plate reader.

### 2.13 Statistics

GraphPad Prism 10 software (version 10.6.1) was used for graph generation and statistical analysis. Data are shown as independent data points representing a single donor or as mean ± standard error of the mean. Sample sizes, technical replicates, and the number of independent experiments are indicated in figure legends and were chosen based on signal robustness, assay variability, and data variation. For each experiment, paired samples from the same donor were used, with samples distributed across the different treatment conditions. Normality was assessed using the Shapiro-Wilk test. Paired statistical tests were selected according to group number and data distribution as detailed in the figure legends. Significance was set at *P* < 0.05. The half-maximal inhibitory or effective concentration (IC_50_ or EC_50_) values were calculated by normalizing data between the smallest and largest means (0% and 100%) and applying non-linear regression.

## 3 Results

### 3.1 ETs trigger inflammation-promoting responses in macrophages

To evaluate the functional response of macrophage subtypes to neutrophil-derived ETs, macrophages were polarized towards states with pro-inflammatory or inflammation-resolving characteristics using GM-CSF + LPS + IFN-γ or M-CSF + IL-4, respectively. Successful cell polarization was confirmed by assessing CD80 and CD163 surface expression and the secretion of IL-6 and IL-10 following LPS stimulation (**Supplementary Figures 3A, B**). Both macrophage phenotypes were exposed to soluble ETs derived from stimulated neutrophils (detailed characterization of the ETs in **Supplementary Figures 2A–F**) using a previously described approach (16–18). The range of ET concentrations used throughout this study did not induce macrophage cell death (**Supplementary Figure 2G**).

Exposure of pro-inflammatory macrophages to ETs resulted in a dose dependent increase in IL-8, TNFα, IFN-γ, IL-1Ra, and CCL3 which, in most independent donors, exceeding the levels induced by 10 ng/mL LPS (**Figure 1A**; **Supplementary Figure 4A**). IL-1β, IL-12p70, IL-6, IL-10 and CCL2 secretion remained unchanged (**Supplementary Figure 4B**). Furthermore, exposure of inflammation-resolving macrophages to ETs resulted in a dose dependent elevated secretion of IL-1Ra and CCL3 in a manner equivalent to levels induced by 10 µg/mL LPS (**Figure 1B**; **Supplementary Figure 4C**). Enhanced TNFα secretion was observed at the highest ET level tested (2000 ng/mL) but the magnitude of response was significantly less than that achieved with LPS. Statistically significant changes were not observed in the secretion of IL-8, IL-6, IL-10, IL-4, and CCL2 (**Supplementary Figure 4D**).

**Figure 1:**
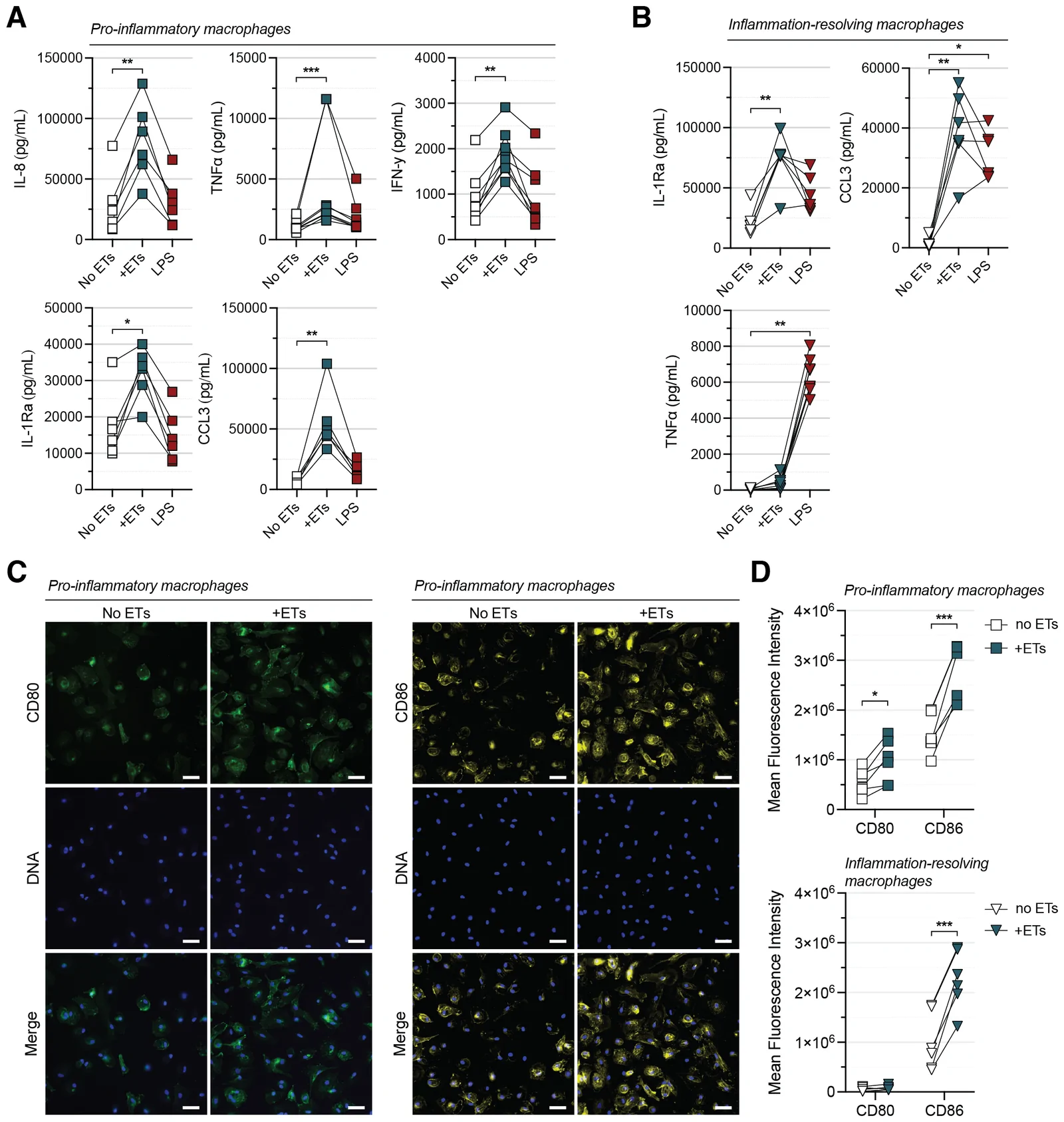
ETs induce pro-inflammatory responses in macrophages. **(A, B)** Cytokine and chemokine levels in supernatants from pro-inflammatory **(A)** and inflammation-resolving **(B)** macrophages following 2000 ng/mL neutrophil-derived ET exposure (+ETs). Macrophages exposed to buffer alone (No ETs) served as negative controls, whereas macrophages stimulated with 10 ng/mL LPS served as positive controls. Two independent experiments using a total of 6–8 donors were performed, with pooled duplicates per donor. **(C)** Representative images of CD80 and CD86 surface expression in pro-inflammatory macrophages with or without ET exposure. Nuclei (blue), CD80 (green), CD86 (yellow). Scale bar = 25 µm. **(D)** Quantification of CD80 and CD86 surface expression in pro-inflammatory and inflammation-resolving macrophages with and without ET exposure. Two independent experiments were performed using a total of 6 donors, with a single measurement per donor. *P < 0.05, **P < 0.01 and *** *P* < 0.001. Statistical tests: Friedman test with Dunn’s multiple comparisons test **(A, B)**, RM one-way ANOVA with Dunnett’s multiple comparisons test **(A)** and two-tailed paired t test **(D)**.

We next assessed the surface expression of costimulatory molecules on both macrophage subsets following exposure to soluble ETs. ET stimulation markedly increased the surface expression of CD80 and CD86 in pro-inflammatory macrophages, whereas inflammation-resolving macrophages only upregulate CD86 surface expression in response to ETs (**Figures 1C, D**; **Supplementary Figures 4E, F**).

Collectively, these findings indicate that soluble ETs elevate the secretion of canonical pro-inflammatory cytokines in both macrophage subtypes and upregulate membrane-associated costimulatory molecules in pro-inflammatory macrophages (CD80 and CD86) and inflammation-resolving macrophages (CD86).

### 3.2 Antibody opsonization of ETs enhances phagocytic activity of macrophages

Having established that ETs induce a pro-inflammatory response in both macrophage subtypes, we next determined whether these macrophages have the capacity to phagocytose soluble ETs. Pro-inflammatory macrophages displayed minimal ET uptake, whereas inflammation-resolving macrophages phagocytosed ETs (**Figures 2A, B**). Opsonization with an anti-citH2A/H4 antibody further increased ET phagocytosis by inflammation-resolving macrophages (**Figures 2C, D**). This was not observed with the corresponding F(ab’)2 fragments, demonstrating that this effect was Fc domain dependent. In addition, opsonization of ETs enabled phagocytosis by pro-inflammatory macrophages also by an Fc domain-mediated mechanism (**Figures 2E, F**). This effect was dependent upon antibody concentration (EC_50_ of 0.60 nM; **Supplementary Figure 5**).

**Figure 2:**
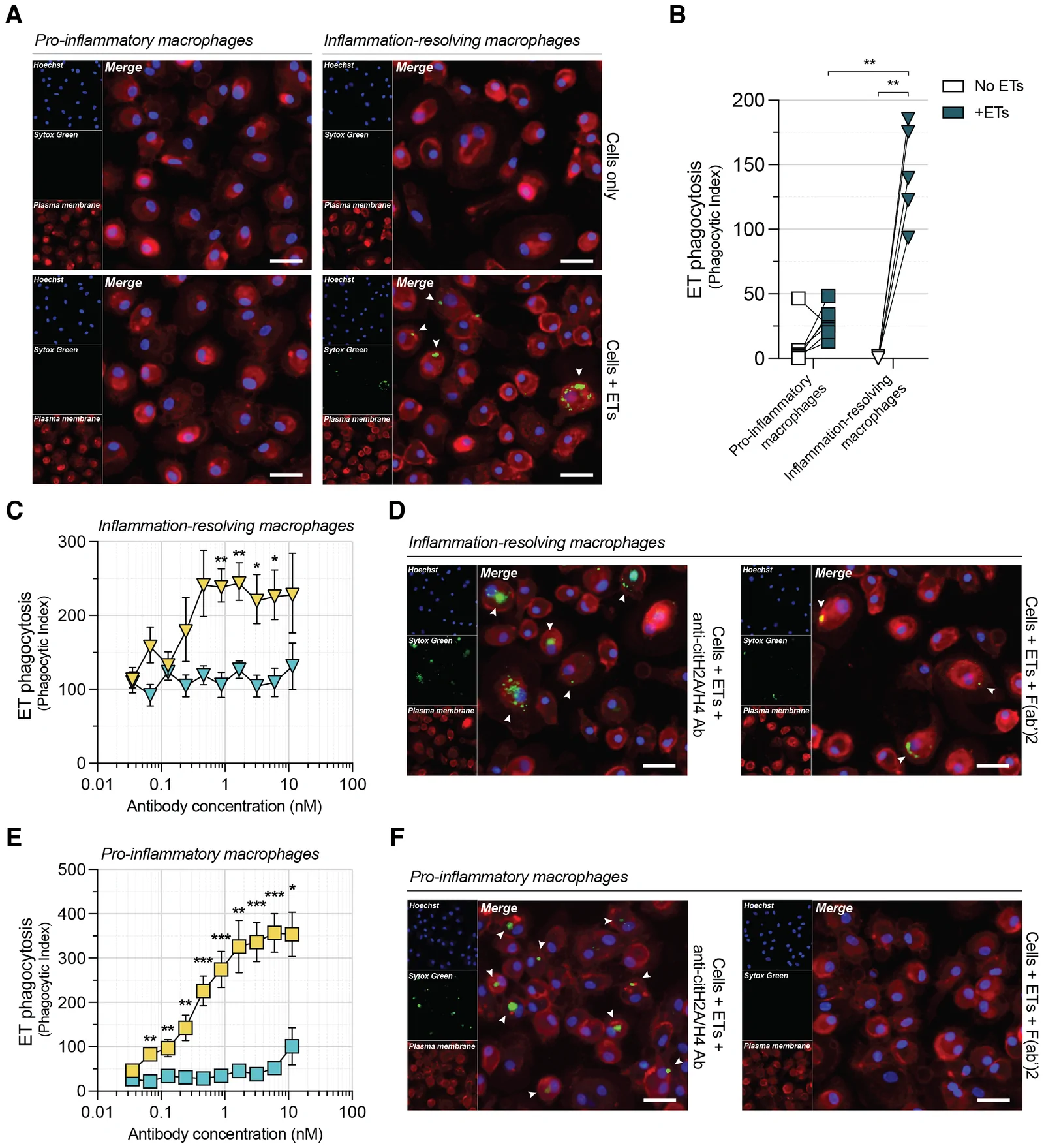
Antibody opsonization enhances macrophage phagocytosis of ETs. **(A)** Representative images of neutrophil-derived ET phagocytosis (arrowheads) by pro-inflammatory and inflammation-resolving macrophages. Nuclei (blue), ETs (green), plasma membrane (red). Scale bar = 25 µm. **(B)** Quantification of ET phagocytosis in the absence of antibody opsonization. **(C–F)** Representative images and quantification of ET phagocytosis by inflammation-resolving **(C, D)** and pro-inflammatory **(E, F)** macrophages following opsonization with increasing concentrations of anti-citrullinated histone H2A and H4 (anti-citH2A/H4) antibody or corresponding F(ab’)2 fragments. Images correspond to 1.66 nM **(D)** or 3.16 nM **(F)** antibody. All experiments were performed in two independent experiments using a total of 5–7 donors, with a single measurement per donor. Statistical comparisons are shown between matched antibody and F(ab’)2 conditions. *P < 0.05, **P < 0.01, and ***P < 0.001. Statistical tests: Two-tailed paired t test **(B, C, E)** and two-tailed Wilcoxon test **(B, E)**.

These findings indicate that the ability to phagocytose native ETs is different across macrophage subsets and that an IgG antibody targeting ETs enables and enhances their phagocytosis in macrophages with different characteristics.

### 3.3 Antibody opsonization of ETs modulates the macrophage immune signaling pathway transcriptome

Given the altered phagocytic behavior of macrophages towards antibody-opsonized ETs, we next examined their transcriptional responses to FcR engagement. To enable this, and noting that immobilization may alter FcR clustering and receptor organization compared with interactions with ETs *in vivo*, the experimental design was modified to study immobilized ETs, allowing the selective analysis of FcR-driven responses independent of transcriptional changes associated with internalization of soluble ETs. PCA showed that exposure to immobilized ETs did not separate either macrophage subtype from their respective untreated (No ETs) or F(ab’)2 treated controls (**Figures 3A, B**). In contrast, anti-citH2A/H4 antibody-opsonized ETs induced a distinct transcriptional signature in both macrophage subsets compared to native ETs, which was more pronounced in pro-inflammatory macrophages (**Figures 3A, B**). Consistent with this, gene set enrichment analysis revealed that significantly enriched pathways were predominantly observed in pro-inflammatory macrophages and were associated with immunomodulatory processes, including TNFα signaling, cytokine-receptor interactions, and mitogen-activated protein kinase (MAPK) signaling (**Figure 3C**). Examination of the most significantly regulated genes across the enriched pathways revealed recurring patterns in both macrophage subsets, characterized by gene expression changes of pro-inflammatory signaling cascades, induction of anti-inflammatory programs, and suppression of genes encoding T- and B-cell-associated cytokines and chemokines (**Figure 3D**; **Supplementary Figure 6A**).

**Figure 3:**
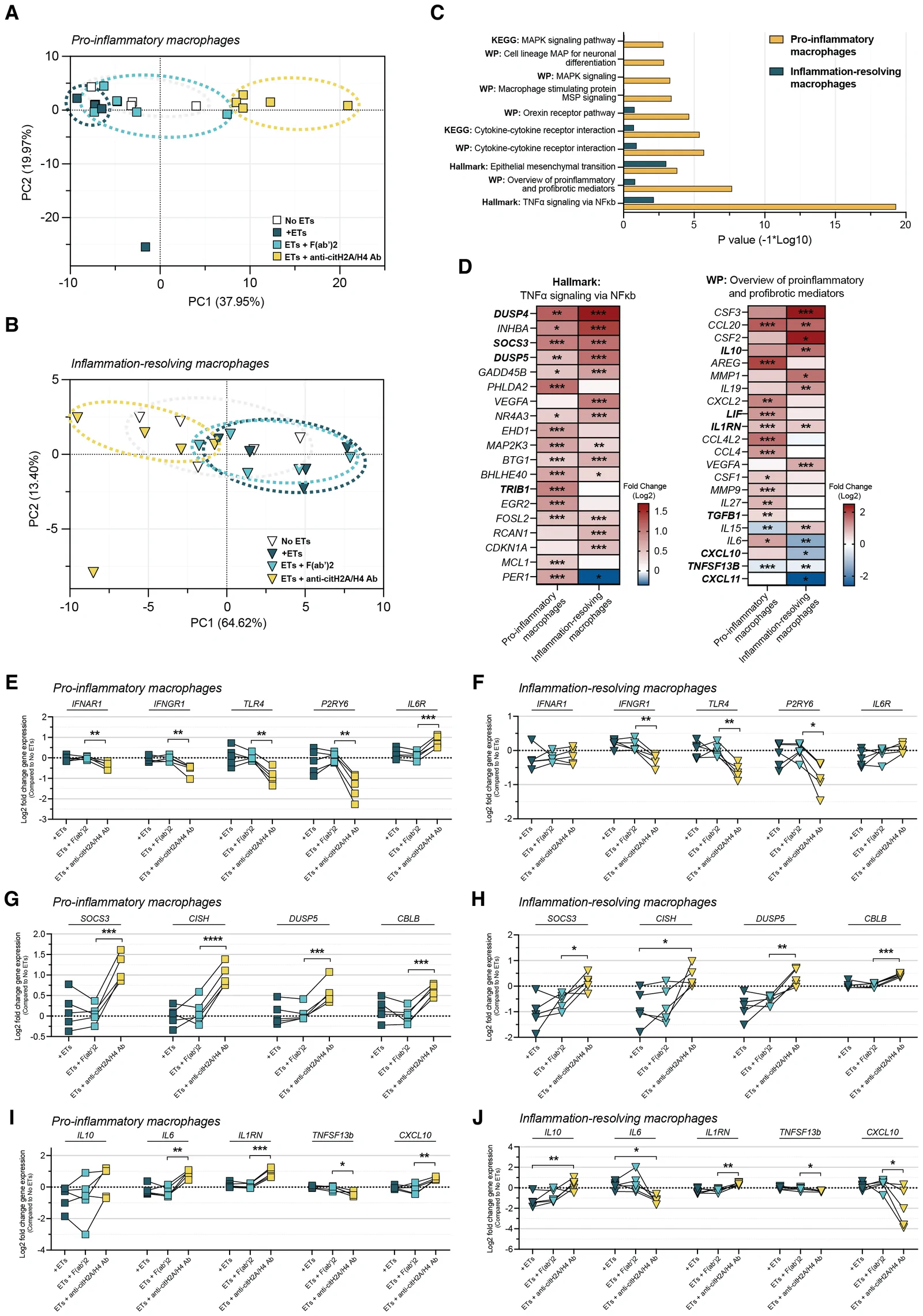
Antibody-opsonized ETs modulate macrophage transcriptional responses. **(A, B)** Principal component analysis of the transcriptomic profiles of pro-inflammatory **(A)** and inflammation-resolving **(B)** macrophages exposed to native neutrophil-derived ETs (+ETs) or ETs opsonized with 166.67 nM anti-citH2A/H4 antibody or corresponding F(ab’)2 fragment. Untreated macrophages (No ETs) served as controls.. Analysis includes genes differentially regulated in at least one condition. **(C)** Top 10 enriched pathways in macrophages exposed to antibody-opsonized ETs relative to native ETs. Gene set enrichment analysis was performed using the HALLMARK, WIKIPATHWAYS (WP), and KEGG collections from the Molecular Signature Database. **(D)** Heatmaps presenting Log2 fold changes in gene expression in pro-inflammatory and inflammation-resolving macrophages following exposure to antibody-opsonized ETs relative to native ETs. **(E–J)** Log2 fold changes of selected genes in pro-inflammatory **(E, G, I)** and inflammation-resolving **(F, H, J)** macrophages exposed to native or opsonized ETs. Data are normalized to untreated controls (dotted line). One experiment was performed using a total of 5 donors, with a single measurement per donor. *P < 0.05, **P < 0.01, ***P < 0.001, and ****P < 0.0001. Statistical tests: Benjamini–Hochberg multiple comparisons test **(D)**, Friedman test with Dunn’s multiple comparisons test **(I, J)**, and RM one-way ANOVA with Dunnett’s multiple comparisons test **(E–J)**.

We next analyzed the changes of individual genes within functional groupings. Following exposure to antibody-opsonized immobilized ETs, both macrophage subsets showed reduced expression of genes (relative to respective controls) encoding interferon gamma receptor 1 (*IFNGR1*) and the membrane-associated pattern-recognition receptors toll-like receptor 4 (*TLR4*) and pyrimidinergic receptor P2Y6 (*P2RY6*) (**Figures 3E, F**). In contrast, interferon alpha receptor 1 (*IFNAR1*), was significantly downregulated in only one of the macrophage subsets. Apart from a reduction in interferon gamma inducible protein 16 (*IFI16*) and elevation in IL-6 receptor (*IL6R*) expression observed in pro-inflammatory macrophages, transcript levels of other pattern-recognition or cytokine receptor were not affected by antibody-opsonized immobilized ETs (**Supplementary Figures 6B, C**).

In both pro-inflammatory and inflammation-resolving macrophages exposed to antibody-opsonized immobilized ETs, increased expression of genes encoding negative regulators of the janus kinase/signal transducer and activator of transcription (JAK/STAT) and MAPK signaling, including suppressor of cytokine signaling 3 (*SOCS3*), cytokine-inducible SH2-containing protein (*CISH*), dual-specificity phosphatase 5 (*DUSP5*) and casitas B-lineage lymphoma b (*CBLB*) was observed (**Figures 3G, H**). In inflammation-resolving macrophages, native immobilized ETs also reduced gene expression of these negative regulators (except CBLB) relative to No ETs (dotted line, **Figure 3H**). In contrast, antibody-opsonized immobilized ETs restored gene expression levels to baseline or elevated them beyond it. Moreover, several cytokine-encoding genes, including *IL10*, *IL6*, *IL1RN*, *TNFSF13B*, and *CXCL10* were significantly regulated following exposure to antibody-opsonized immobilized ETs in one or both macrophage subsets (**Figures 3I, J**).

Together, these findings demonstrate that antibody opsonization of ETs elicits a transcriptional modulatory effect in both macrophage subtypes, broadly dampening pro-inflammatory signaling pathways through reduced expression of pro-inflammatory receptors and increased expression of negative regulators and anti-inflammatory mediators.

### 3.4 Exposure to antibody-opsonized ETs attenuates LPS and IFN-γ-mediated macrophage surface expression of costimulatory molecules

Based on the observation that antibody opsonization of ETs downregulated expression of genes encoding TLR4 and IFN-γ receptor (**Figures 3E, F**), which are both critical for macrophage polarization for a phenotype consistent with pro-inflammatory characteristics, we examined whether antibody-opsonized immobilized ETs modulate pro-inflammatory macrophage activity. To test this, macrophages were cultured with GM-CSF for 6 days followed by exposure to native ETs or anti-citH2A/H4 antibody-opsonized ETs, and then stimulation with LPS and IFN-γ to induce a pro-inflammatory phenotype. In these macrophages, exposure to antibody-opsonized ETs reduced surface expression of co-stimulatory molecules CD80 and CD86, effects that were not observed with the corresponding F(ab’)2 fragment (**Figures 4A, B**). Furthermore, assessment of cytokine release showed that antibody-opsonized ETs efficiently decreased secretion of IL-12p70, CXCL9 and IL-6, whereas TNFα reduction was more variable across donors (**Figure 4C**). Levels of IL-1β, IL-8, IL-10, IL-1Ra, IFN-γ, and CCL3 remained unchanged, with exception of CCL2 showing a slight increase (**Supplementary Figure 7**).

**Figure 4:**
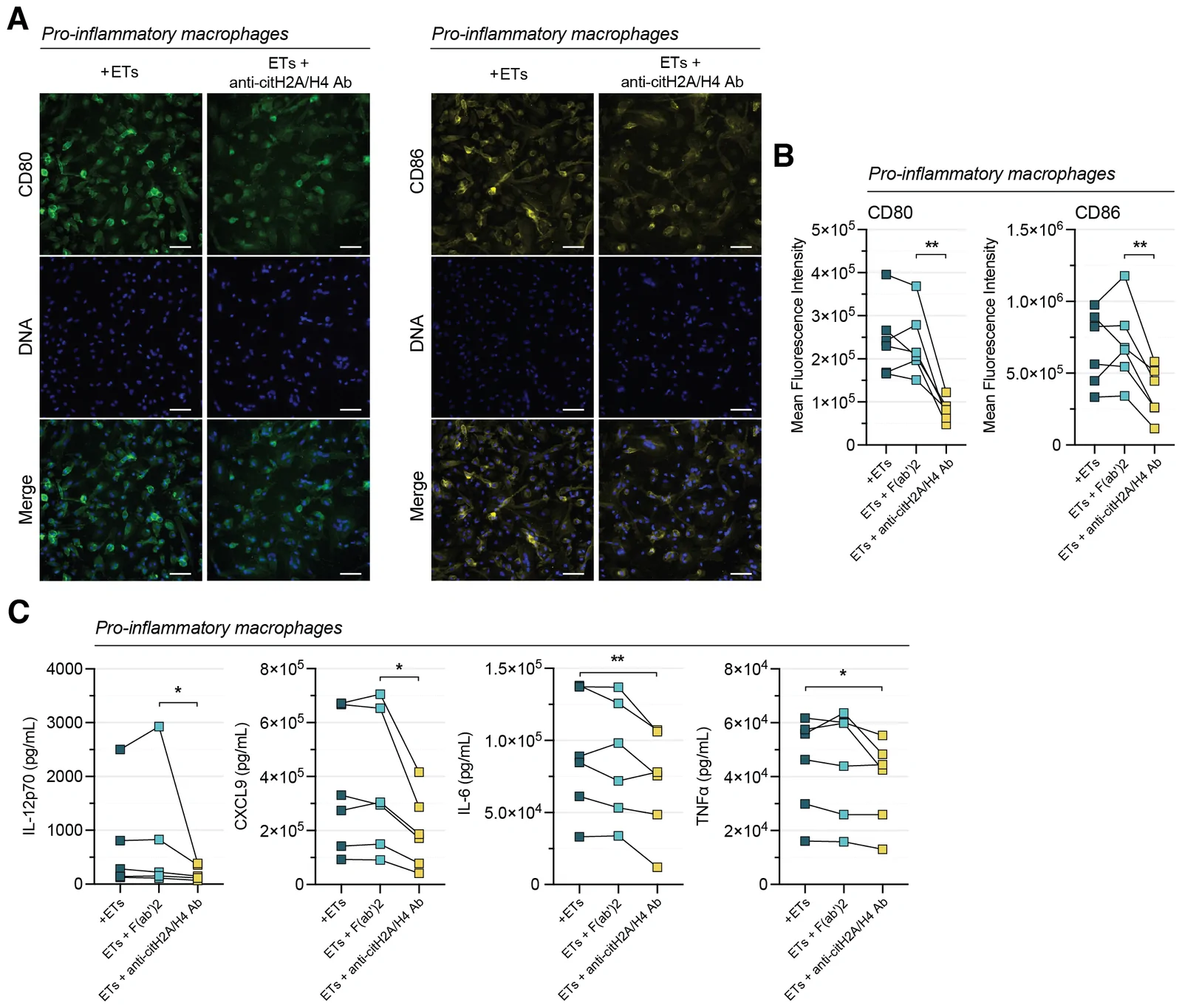
Antibody-opsonized ETs attenuate LPS and IFN-γ-mediated macrophage surface expression of costimulatory molecules. **(A)** Representative images of CD80 and CD86 surface expression in pro-inflammatory macrophages exposed to native neutrophil-derived ETs (+ETs) or ETs opsonized with 166.67 nM anti-citH2A/H4 antibody following stimulation with LPS and IFN-γ. Nuclei (blue), CD80 (green), CD86 (yellow). Scale bar = 50 µm. **(B)** Quantification of CD80 and CD86 surface expression in these macrophages. Two independent experiments were performed using a total of 6 donors, with a single measurement per donor. **(C)** Levels of IL-12p70, CXCL9, IL-6, and TNFα in supernatants from these macrophages. Two independent experiments were performed using a total of 5–6 donors, with pooled duplicates per donor. *P < 0.05 and **P < 0.01. Statistical tests: RM one-way ANOVA with Dunnett’s multiple comparisons test **(B, C)** and Friedman test with Dunn’s multiple comparisons test **(C)**.

Together, these functional effects confirm the transcriptional modulation of pro-inflammatory macrophages and indicate that antibody-opsonized ETs suppress macrophage secretion of cytokines and chemokines that engage T cells and surface expression of T cell-associated costimulatory molecules.

### 3.5 Antibody-opsonized ETs attenuate TLR-MAPK-dependent CXCL9 and CXCL10 release in macrophages

We next assessed the functional consequences of antibody opsonization of immobilized ETs for inflammation-resolving macrophages. Macrophages were pre-exposed to either native or anti-citH2A/H4 antibody-opsonized ETs followed by stimulation with LPS, a potent TLR4 agonist that activates the MAPK signaling cascade. CXCL9 served as a principal readout of MAPK-dependent TLR4 signaling, an approach validated by inhibition of CXCL9 secretion with the MAPK2 inhibitor Zunsemetinib (**Supplementary Figure 8A**). In inflammation-resolving macrophages, LPS significantly stimulated CXCL9 release which was unaffected by pre-exposure to native ETs indicating that ETs do not modulate LPS responsiveness (**Figure 5A**). Strikingly, pre-exposure of the same macrophages to antibody-opsonized ETs markedly inhibited LPS-induced CXCL9 production in a manner dependent on antibody concentration (IC_50_ of 2.22 nM; **Figure 5B**; **Supplementary Figure 8B**). This suppressive effect was not observed with the corresponding F(ab’)2 fragment or the anti-citH2A/H4 antibody alone indicating that immune complex formation and FcR engagement are required for suppression. Consistent with this, blockade of FcγRI or FcγRII significantly attenuated the suppression of CXCL9 release, with the effect of FcγRIII blockade failing to achieve statistical significance (**Figure 5C**; **Supplementary Figure 8C**). Furthermore, inflammation-resolving macrophages exhibit high surface expression of FcγRII and modest surface expression of FcγRI (**Supplementary Figure 8D**), providing a potential mechanistic basis for the involvement of both receptors.

**Figure 5:**
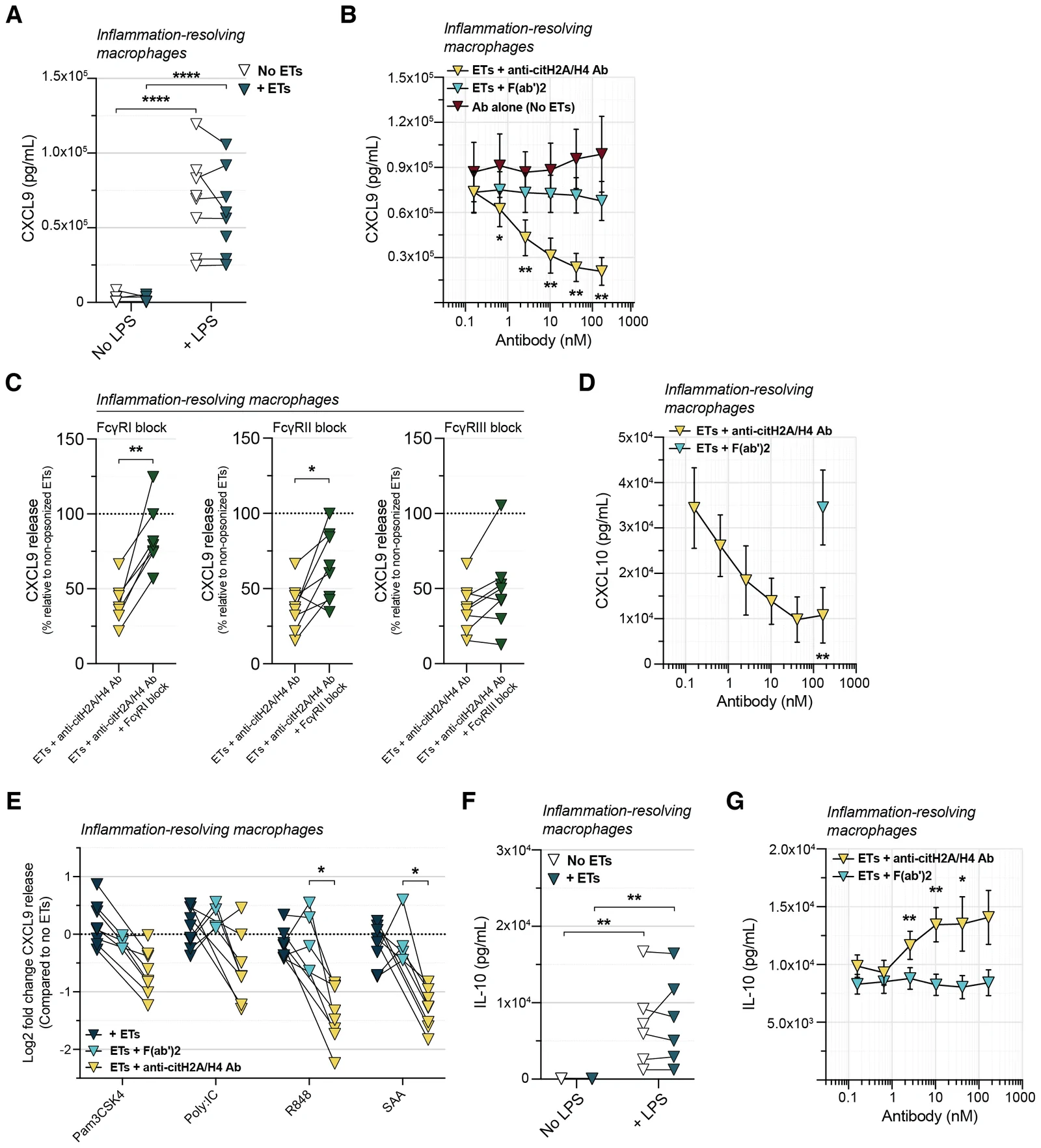
Antibody-opsonized ETs attenuate TLR-MAPK-dependent CXCL9 and CXCL10 release in macrophages. (A-G) Inflammation-resolving macrophages pre-exposed to empty wells (No ETs), native ETs (+ ETs), or ETs opsonized with indicated concentrations of anti-citH2A/H4 antibody or corresponding F(ab’)2 fragments, followed by LPS stimulation. **(A, B)** CXCL9 levels in the supernatants of these macrophages. Two independent experiments were performed using a total of 3-8 donors, with a single measurement per donor. **(B)** Antibody alone without ETs (Ab alone) served as a control. **(C)** Relative CXCL9 levels in the supernatant of these macrophages pre-exposed to ETs opsonized with 42.32 nM anti-citH2A/H4 antibody in the presence or absence of FcɣRI, FcγRII, or FcγRIII block, followed by LPS stimulation. Data is normalized to CXCL9 levels released by macrophages pre-exposed to native ETs (dotted line). Two independent experiments were performed using a total of 7-8 donors, with a single measurement per donor. **(D)** CXCL10 levels in the supernatants of these macrophages pre-exposed to opsonized ETs with indicated concentrations of anti-citH2A/H4 antibody or corresponding F(ab’)2 fragments, followed by LPS stimulation. Two independent experiments were performed using a total of 4-8 donors, with a single measurement per donor. **(E)** Log2 fold changes in CXCL9 secretion in the supernatant of inflammation-resolving macrophages stimulated with other TLR agonists, including Pam3CSK4, Poly(I:C), R848, or serum amyloid a (SAA). Two independent experiments were performed using a total of 4-8 donors, with a single measurement per donor. Data are normalized to TLR stimulation in the absence of ETs (dotted line). **(F, G)** IL-10 levels in the supernatants from inflammation-resolving macrophages stimulated with LPS. Two independent experiments were performed using a total of 4-6 donors, with technical duplicates per donor. Statistical comparisons in **(B, D, E , G)** are between matched antibody and F(ab’)2 fragment conditions.

As observed for CXCL9, reduced CXCL10 secretion was also seen when inflammatory-resolving macrophages were pre-exposed to antibody-opsonized ETs following LPS stimulation. This was also dependent upon the antibody concentration (IC_50_ of 1.49 nM; **Figure 5D**; **Supplementary Figure 8E**).

The possibility that the suppressive effect of antibody-opsonized ETs might extended beyond TLR-MAPK activation by LPS/TLR4 was assessed by stimulation of inflammation-resolving macrophages with distinct TLR agonists. A CXCL9 secretion response to R848 and SAA was similarly inhibited by anti-citH2A/H4 antibody binding to ETs in an Fc domain-dependent manner, while a trend toward reduced CXCL9 release was observed following stimulation with Pam3CSK and Poly(I:C) (**Figure 5E**). In contrast, pro-inflammatory macrophages failed to show LPS-induced enhancement of CXCL9 due to prior LPS or IFN-γ exposure during polarization (**Supplementary Figure 8F**) (33) and antibody-opsonized ETs did not diminish CXCL9 levels (**Supplementary Figure 8G**), suggesting that fully activated pro-inflammatory macrophages, previously exposed to LPS, are refractory to this regulatory mechanism.

Following LPS stimulation of inflammation-resolving macrophages, IL-10 secretion was also increased in a manner unaffected by native ETs (**Figure 5F**). However, consistent with the induction of anti-inflammatory transcriptional programs, antibody-opsonized ETs strongly enhanced IL-10 production in an antibody concentration-dependent manner (EC_50_ of 2.86 nM; **Figure 5G**; **Supplementary Figure 8H**).

Together, these findings demonstrate that antibody-opsonized ETs enhance IL-10 secretion and attenuate MAPK-dependent signaling in inflammation-resolving macrophages as characterized by reduced T cell-associated chemokine release in response to ligands with a range of different TLR specificities.

## 4 Discussion

This study demonstrates that discrete macrophage subsets, consistent with pro-inflammatory (GM-CSF + LPS + IFN-γ differentiated) or inflammation-resolving (M-CSF + IL-4 differentiated) characteristics, exhibit distinct responses to engagement with neutrophil-derived ETs, offering insight into the dynamics between ETs and the inflammatory status of tissue. ETs induce inflammation-promoting responses in macrophages and ET opsonization with an anti-citH2A/H4 antibody adjust macrophage responses across subsets toward a new, less inflamed equilibrium, by enhancing ET phagocytosis, modulating macrophage gene expression, and attenuating pro-inflammatory signaling. These findings support the existence of an IgG Fc domain-dependent mechanism that modulates inflammatory responses to ETs and suggests macrophage subtypes are less responsive to pro-inflammatory triggers. In addition, macrophages can express inflammation resolution-associated characteristics when engaged by antibody-opsonized ETs distinct from the inflammation responses shown otherwise to native ETs. While these findings provide mechanistic insight into macrophage-ET interactions, their relevance *in vivo* remains to be established and will require validation in disease-relevant experimental systems. Such studies will be important to determine the therapeutic potential of targeting ETs in diseases in which they contribute to pathogenesis.

Accumulating evidence implicates ETs in a wide range of pathological processes, including acute inflammatory conditions such as sepsis (34), sterile inflammatory disorders such as stroke and thrombosis (13), autoimmune diseases including RA and systemic lupus erythematosus (SLE) (13, 35), autoinflammatory conditions such as HS (36, 37) and cancer (13, 38). Consistent with these observations, our data suggest that ET-associated pathology and inflammation are at least in part, driven by ET-induced pro-inflammatory activation of macrophages, important effector cells in the pathogenesis of these diseases. Among ET components, citrullinated histones have emerged as key pathogenic mediators, capable of driving disease progression and tissue damage by amplifying immune activation (39–41) and inducing cytotoxicity (42). Consequently, targeting the citrullinated histone component of ETs may represent a promising, novel therapeutic strategy.

In support of this, research indicates that a humanized monoclonal antibody targeting citrullinated histone H3 can restore macrophage function in an animal model of sepsis (41). Furthermore, the anti-arthritic activity of an IgG2b monoclonal antibody targeting murine citrullinated α-enolase is dependent upon Fc receptor-mediated macrophage engagement (43). Finally, targeting the citrullinated forms of histones H2A and H4 with the human monoclonal antibody used in this study (CIT-013), has been previously shown to inhibit ET formation from neutrophils (28) and eosinophils (29) and to result in Fc domain dependent anti-inflammatory effect in a mouse model of arthritis (27). This present study extends the established anti-inflammatory activity of CIT-013 by demonstrating that antibody-mediated opsonization of ETs modulates macrophages in an Fc domain-dependent manner to attenuate their responses to inflammatory stimuli. Evaluation of the therapeutic relevance of this mechanism will require studies focusing on macrophage phenotypes in the inflamed tissue of inflammatory diseases before and after treatment. Ongoing phase 2a clinical studies with CIT-013 in RA and HS (NCT06567470, NCT06993233) will provide insights into the therapeutic impact of antibody-mediated opsonization of ETs.

Efficient clearance of ETs requires DNase-mediated fragmentation followed by phagocytic uptake and intracellular degradation by macrophages (16, 17, 44). Our data indicate that ET clearance is not uniformly efficient across macrophage subsets, consistent with previous reports demonstrating inferior phagocytic capacity of pro-inflammatory macrophages relative to inflammation-resolving macrophages (45, 46) with the former being the dominant macrophage type at sites of chronic inflammation (47). Together, these findings suggest that an imbalance of macrophage subtypes in chronically inflamed tissue contributes to inefficient ET clearance, local ET accumulation, tissue damage, exacerbation of auto-antigen exposure and inflammation. In line with this concept, elevated ET levels resulting from impaired clearance have been associated with clinical manifestations in several IMIDs, including SLE (48, 49), anti-phospholipid syndrome (50), HS (51) and RA (52). Collectively, these observations highlight ET clearance as an important determinant of inflammation resolution.

Antibody opsonization of ETs enhances their phagocytic uptake by inflammation-resolving and pro-inflammatory macrophages, consistent with the well-established role of FcR engagement in promoting phagocytosis (53). Beyond phagocytosis, engagement of FcγRs by immune complexes can elicit broad cellular responses, with Src family kinase activation triggering multiple branched signaling transduction pathways that regulate intracellular calcium, cytoskeletal rearrangement, cell-cycle progression, transcriptional responses, and mediator release (54). Importantly, the functional outcome of FcγR engagement is dependent on the cellular and inflammatory context. Crosstalk between FcγR signaling and pathways activated by other receptors, including TLRs, integrins, and interferon receptors (55–57), can further shape the magnitude and nature of macrophage responses. The context dependency is consistent with the findings reported here that, in the absence of an additional stimulus antibody-opsonized ETs induced modest increases in pro-inflammatory gene expression while exposure to antibody-opsonized ETs in the context of TLR stimulation attenuates macrophage pro-inflammatory responses.

The observation that antibody-ET immune complexes attenuate pro-inflammatory signaling contrasts with the canonical association of immune complexes with development, progression and severity of autoimmune diseases (58, 59). Immune complexes in autoimmune diseases are generated through targeting self-antigens and induce persistent immune reactions against host tissue. In multiple autoimmune diseases, excessive immune complex accumulation has been reported to result in aberrant FcγRI activation (60) with abnormal Fc domain glycosylation recognized as an important determinant of such outcomes (61, 62). Autoantibodies associated with inflammatory disorders, including RA, also preferentially lack galactose and sialic acid residues compared with non-autoreactive IgG (61, 63). Indeed, anti-citrullinated protein autoantibodies which are predictors of arthritis development and contribute to synovial inflammation (58, 64) exhibit a progressive loss of Fc galactosylation and altered fucosylation prior to disease onset. These changes correlate with increased systemic inflammation and are predicted to enhance FcγR engagement (65–67). The observations reported in this manuscript, support a model in which the pro- or anti-inflammatory consequence of macrophage engagement by immune complexes in inflamed tissue are not determined solely by the presence of autoantibodies.

This study has limitations. Firstly, experiments with macrophages differentiated from peripheral blood monocytes of healthy donors cannot recapitulate the complexity of macrophage biology within diseased tissues. In inflammatory diseases, tissue-resident and infiltrating macrophages are shaped by local microenvironmental cues and may harbor FcR polymorphisms and copy number variations that could influence their responses to antibody-ET immune complexes (68, 69). Secondly, the use of immobilized ETs in functional and transcriptomic experiments provides a controlled system to investigate macrophage responses, however immobilization on a plastic surface most likely does not reflect the presentation and spatial organization of ETs encountered by macrophages in tissues. Immobilized immune complexes may promote increased FcR clustering and thereby influencing downstream signaling and macrophage activation. Consequently, validation in disease-relevant experimental systems will be important to establish the broader physiological relevance of these findings. Thirdly, a direct causal relationship between enhanced ET phagocytosis and the subsequent transcriptional and functional changes in macrophages has not been established. Although pharmacological inhibition of phagocytosis could be an approach to address this question, commonly used inhibitors such as cytochalasin D disrupt actin polymerization and alter macrophage signaling and cytokine production independent of its effect on phagocytosis (70, 71). Such experimental approaches are likely to be difficult to interpret, highlighting the need for strategies to interference with ET internalization and thereby the determine the extent to which phagocytosis contributes to the observed macrophage responses. A final limitation of the study is that the current experiments do not fully establish whether the observed macrophage responses are specific to anti-citH2A/H4 antibody-mediated ET opsonization or may, in part, reflect a generic response to IgG-opsonized ETs. Although comparator antibodies were evaluated, substantial differences in ET-binding efficiency prevented a well-controlled functional comparison at equivalent antibody occupancy (data not shown). Further studies using appropriately matched ET-binding antibodies would be required to establish the contribution of antigen specificity to the observed macrophage responses.

## Data Availability

All raw numerical data supporting the findings of this study are available from the authors, along with unique reagents referenced in this Article. The raw and processed RNA-seq data generated in this study have been deposited in the Gene Expression Omnibus (GEO) database under accession number GSE332957.
