# Supplementary material for: Antibody-opsonized extracellular traps modulate macrophage responses and attenuate their T cell-associated inflammatory and costimulatory characteristics
Source: Front Immunol. 2026 Sep 29;17:1867666. doi: 10.3389/fimmu.2026.1867666 (PMC13657913; doi:10.3389/fimmu.2026.1867666)
Supplement: Supplementary file 1 [file SupplementaryFile1.pdf]

frontiers

The International Society for the Advancement of Cytometry (ISAC) has highlighted the importance of including comprehensive methodological information to ensure data reproducibility and reliability. In line with this, Frontiers in Immunology now requires authors to submit a checklist for manuscripts that involve flow or mass cytometry. This checklist helps standardize the reporting process, improving the quality and transparency of published data. By doing so, we support scientific progress, making it easier for other researchers to replicate and validate experiments.

This form should be submitted with any manuscripts using flow or mass cytometry.

Sample/specimen/material description

- [ ] Total blood
- [ ] PBMCs
- [ ] Organ digests

Other Monocytes isolated from the PBMC fraction

Did the samples suffer any treatment before or after incubation with the antibodies?

- [ ] Drug ___________________________________
- [ ] Cell permeabilization ______________________
- [x] Dye Fixable Viability Dye eFluor 506
- [ ] Propidium iodine
- [ ] Not applicable

Other Human TruStain FcX

Instrument and antibodies

Name of the Cytometer BD FACSCantoTM II or FACSymphony™ A3 using FACSDiva3

Antibodies and targets	Fluorochrome/ Metal	Catalog number/Company
e.g. anti-CD4	FITC	Cat. XXX/ XXX ltd.
anti-CD14	APC-H7	BD, 560180

anti-CD45	APC	BioLegend, 304037
anti-CD41	PE	BD, 303706
Fixable Viability Dye	eFluor 506	Invitrogen, 65-0866-14
anti-Fcgamma receptor		Biolegend, 305047
anti-Fcgamma receptor		StemCell Technologies,
anti-Fcgamma receptor		Invitrogen, 16-0166-85
Mouse anti-hen egg		CrownVivo, C0005
Mouse anti-hen egg		CrownVivo, C0008
goat anti-mouse IgG	Alexa Fluor Plus 488	Invitrogen, A32723

Data analyses

1. Name of the software FlowJo (v10.10.0)
2. Reference gating strategy in the manuscript or supplementary material

Gating strategy in (eg Figure X) Supplementary Figure 1
