# Supplementary material for: Antibody-opsonized extracellular traps modulate macrophage responses and attenuate their T cell-associated inflammatory and costimulatory characteristics
Source: Front Immunol. 2026 Sep 29;17:1867666. doi: 10.3389/fimmu.2026.1867666 (PMC13657913; doi:10.3389/fimmu.2026.1867666)
Supplement: Supplementary file 2 [file SupplementaryFile2.pdf]

Antibody-opsonized extracellular traps modulate macrophage responses and attenuate their T cell-associated inflammatory and costimulatory characteristics

Annemarie Kip[1], Eline Zwiers[1], Kelsy Waaijenberg[1], Wynand Alkema[2], Eric Meldrum[1], Maarten van der Linden[1]

[1]*Citryll B.V., Oss, The Netherlands*

[2]*Tenwise B.V., Leiden, The Netherlands*

-

Supplementary figures

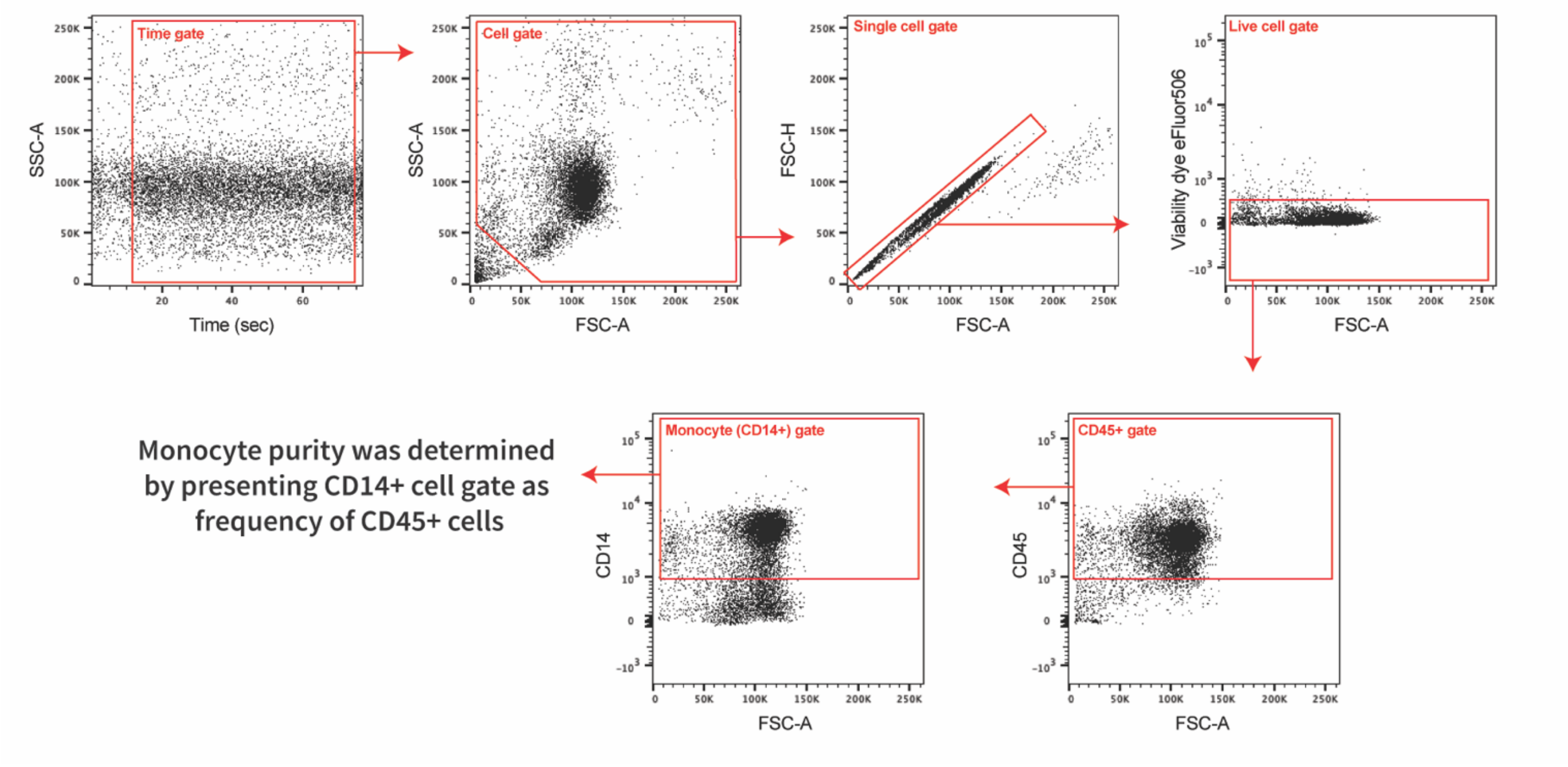

Supplementary Figure 1

Assessment of monocyte purity following isolation from peripheral blood of healthy volunteers. Representative flow cytometry gating strategy used to assess monocyte purity, including time gate, cell gate based on forward scatter (FSC)/side scatter (SSC), single cell gate based on FSC area and height (FSC-A/FSC-H), live cell gate based on viability dye staining, $CD45^{+}$ leukocyte gate, and $CD14^{+}$ monocyte gate. Monocyte purity was determined as the frequency of $CD14^{+}$ monocytes among $CD45^{+}$ cells. Single measurements were performed once for each donor.

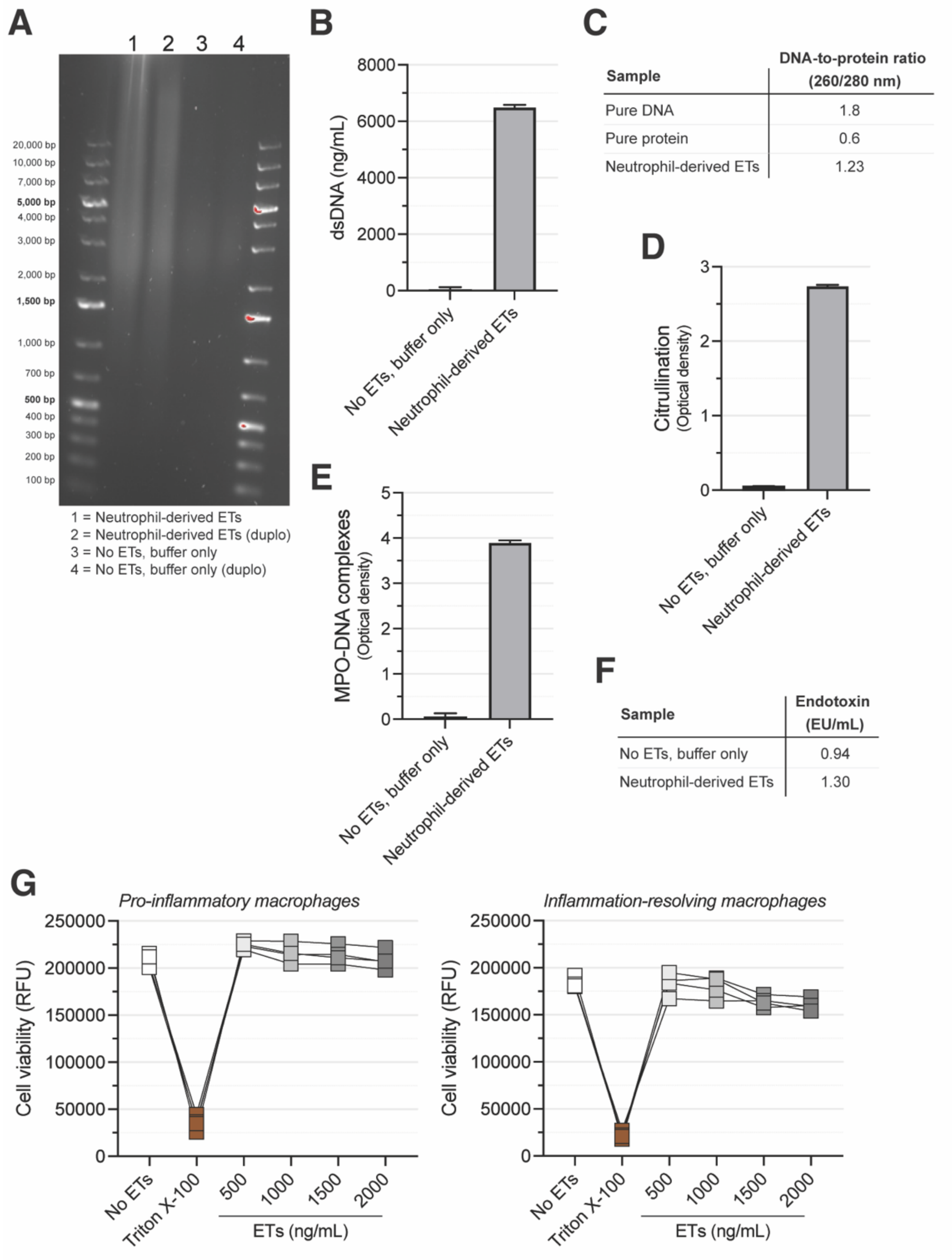

Sample	DNA-to-protein ratio (260/280 nm)
Pure DNA	1.8
Pure protein	0.6
Neutrophil-derived ETs	1.23

Sample	Endotoxin (EU/mL)
No ETs, buffer only	0.94
Neutrophil-derived ETs	1.30

Supplementary Figure 2

Characterization of neutrophil-derived ETs. **(A)** Agarose gel electrophoresis of pooled ETs harvested from stimulated neutrophils derived from 6 donors. ETs were digested with S7 nuclease, resulting in fragments ranging in size from approximately 700 to >20,000 base pairs. **(B,C)** Measurement of dsDNA concentration (B) and DNA-to-protein ratio (C) in the pooled ET preparation. **(D,E)** Detection of citrullinated nucleosomes (D) and MPO-DNA complexes (E) in the pooled ET preparation. **(F)** Quantification of endotoxin levels in the pooled ET preparation. **(G)** Assessment of macrophage viability following exposure to increasing concentrations of native ETs. Macrophages exposed to buffer alone (No ETs) served as negative controls, whereas macrophages treated with 0.01% Triton X-100 served as positive controls. Measurements in (A-G) were performed a single experiment with technical duplicates. RFU, relative fluorescence units.

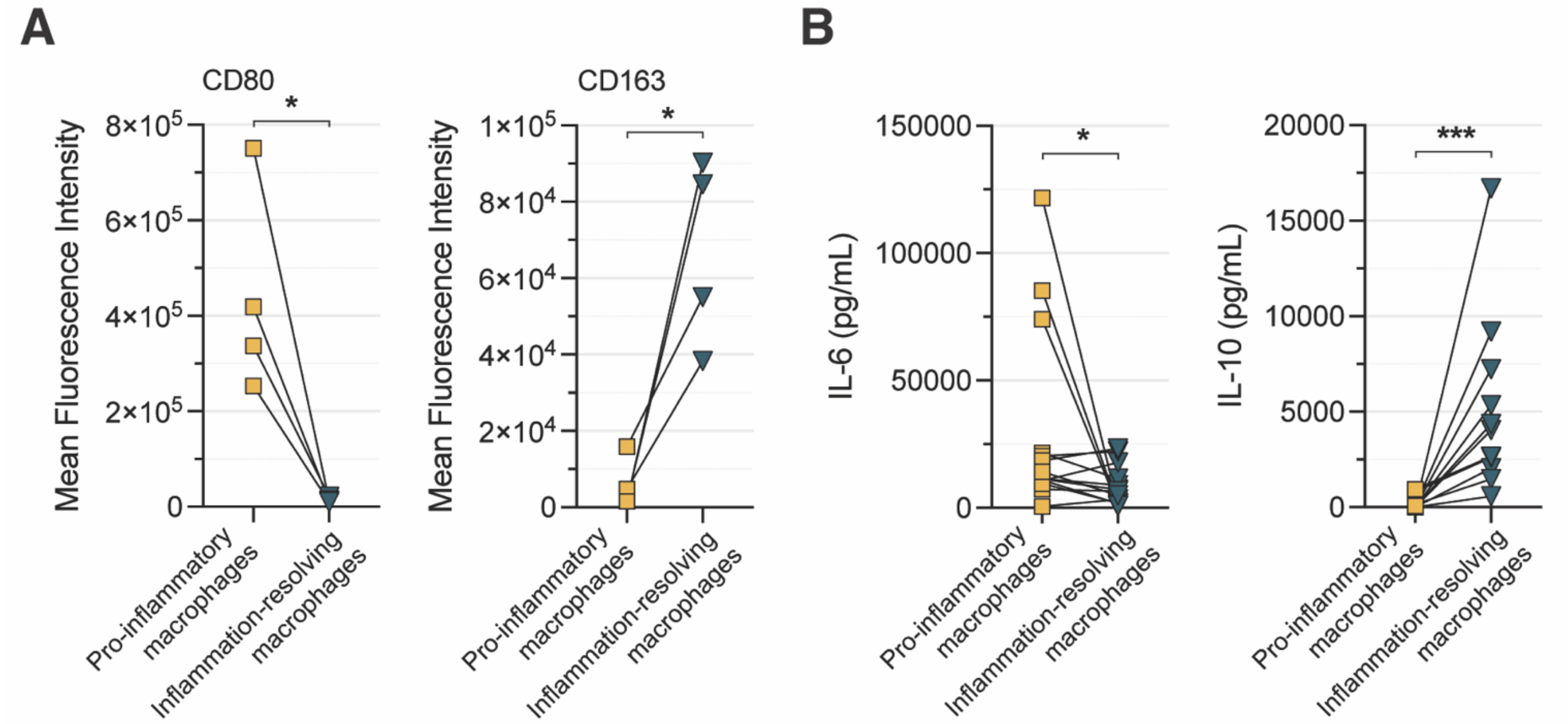

Supplementary Figure 3

Characterization of macrophage cell polarization. **(A)** Immunofluorescence microscopy analysis of CD80 and CD163 surface expression in pro-inflammatory and inflammation-resolving macrophages. Two independent experiments were performed using a total of 4 donors, with technical duplicates per donor. **(B)** IL-6 and IL-10 levels in supernatants from pro-inflammatory and inflammation-resolving macrophages following LPS stimulation. Four independent experiments were performed using a total of 11-14 donors, with technical duplicates per donor. *P<0.05, and ***P<0.001. Statistical tests: Two-tailed paired t test (A) and two-tailed Wilcoxon test (B).

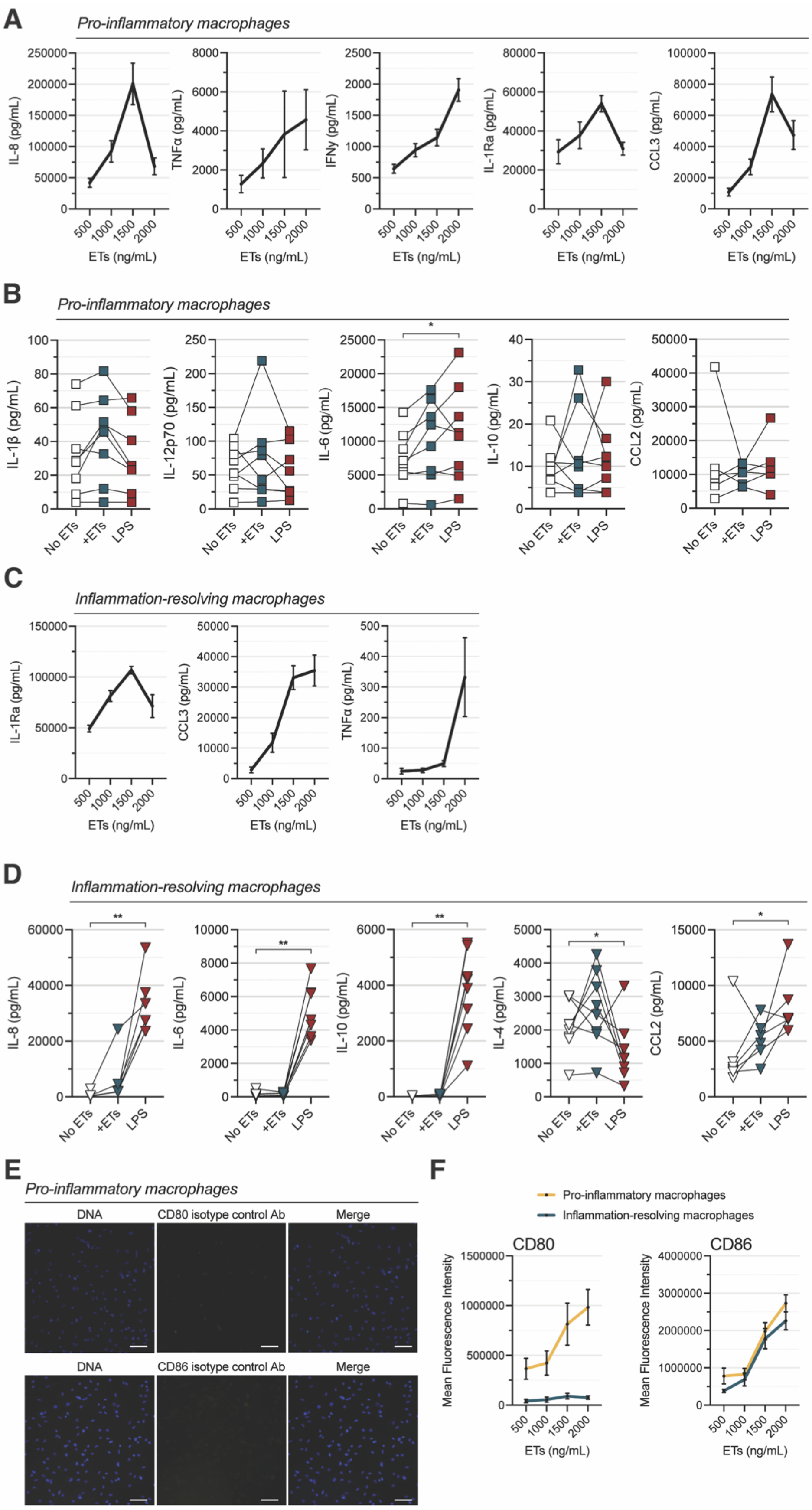

Supplementary Figure 4

ETs induce cytokine and chemokine secretion in macrophages. **(A-D)** Cytokine and chemokine concentrations in supernatants from pro-inflammatory (A,B) and inflammation-resolving (C,D)

macrophages following exposure to increasing concentrations of neutrophil-derived ETs (A,C) or to 2000 ng/mL ETs (+ETs). Macrophages exposed to buffer alone (No ETs) served as negative controls, whereas macrophages stimulated with 10 ng/mL LPS served as positive controls. Two independent experiments using a total of 6-8 donors were performed, with pooled duplicates per donor. **(E)** Representative images of CD80 and CD86 isotype control antibodies. Nuclei (blue). Scale bar = 25 µm. **(F)** Quantification of CD80 and CD86 surface expression in pro-inflammatory and inflammation-resolving macrophages following exposure to increasing concentrations of ETs. Two independent experiments were performed using a total of 6 donors, with a single measurement per donor. *P<0.05 and **P<0.01. Statistical tests: RM one-way ANOVA (B,D) and Friedman test (D).

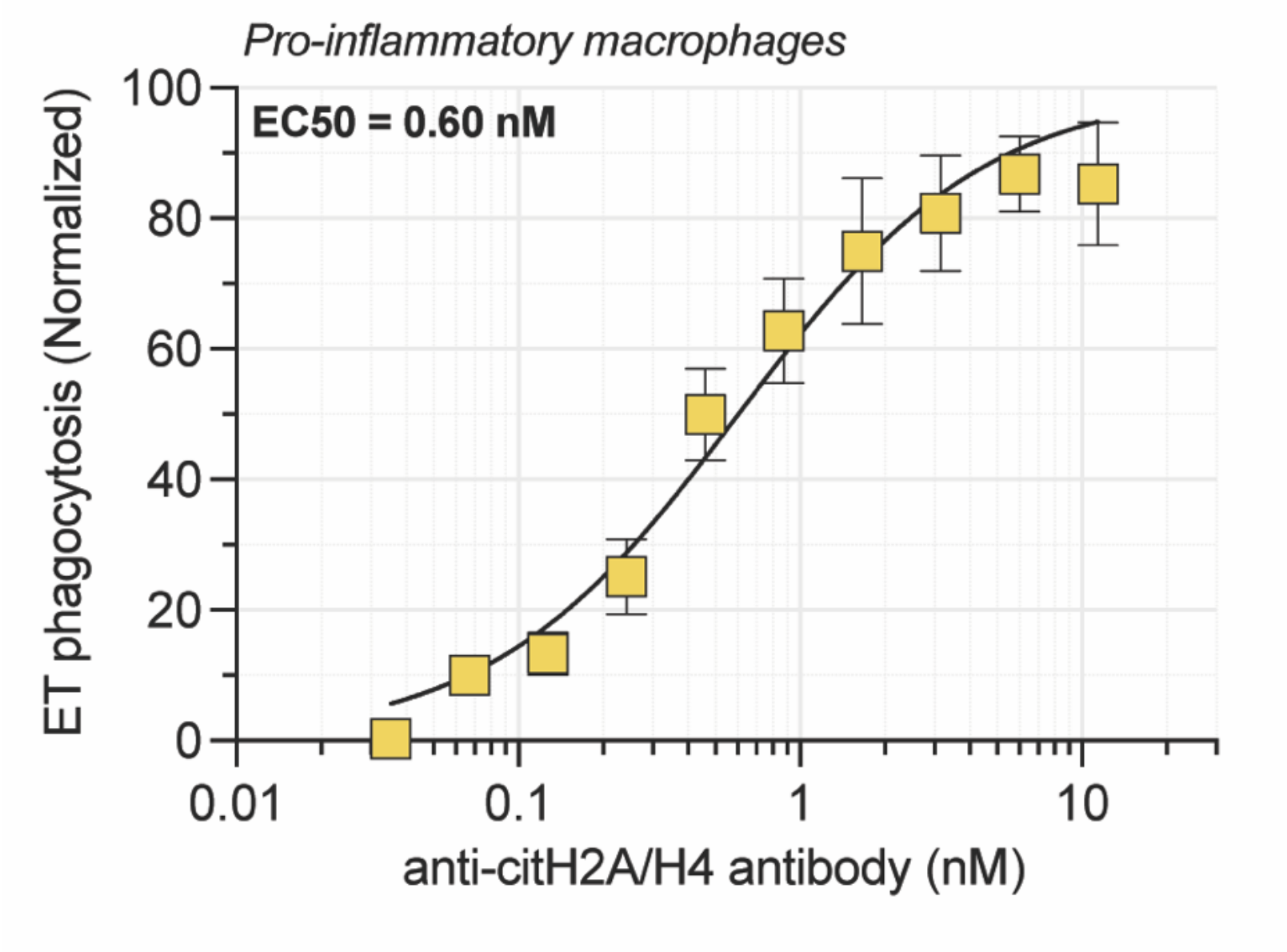

Supplementary Figure 5

ET phagocytosis by macrophages. Normalized phagocytosis of neutrophil-derived ETs in pro-inflammatory macrophages following opsonization with increasing concentrations of anti-citrullinated histone H2A/H4 (anti-citH2A/H4) antibody. Two independent experiments using a total of 7 donors, with a single measurement per donor.

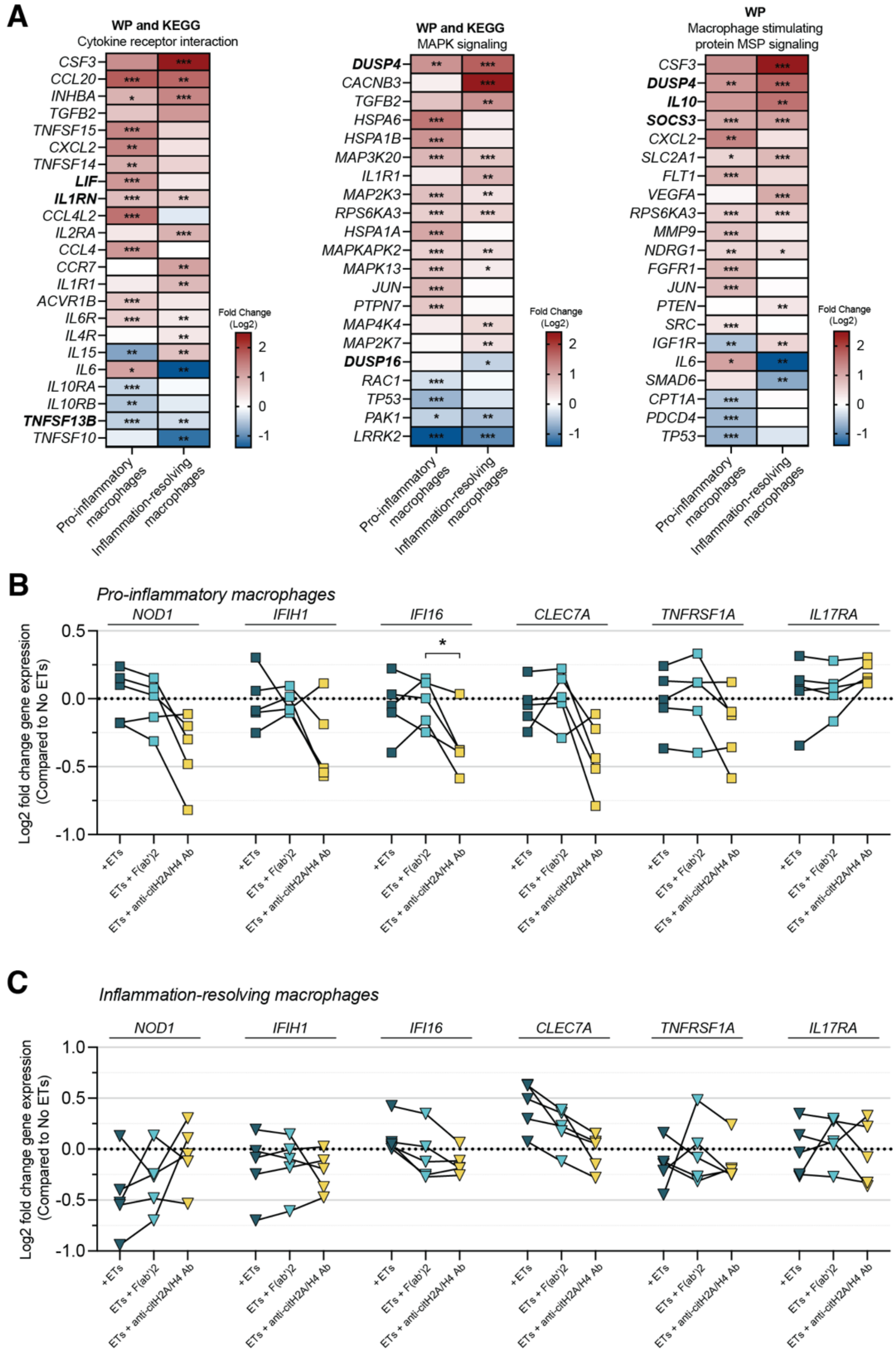

Supplementary Figure 6

Transcriptional modulation of macrophages induced by antibody-opsonized ETs. **(A)** Heatmap presenting Log2 fold changes of selected genes in macrophages exposed to native neutrophil-derived ETs versus antibody-opsonized ETs. **(B,C)** Log2 fold changes of selected genes in pro-inflammatory (B) and inflammation-resolving (C) macrophages exposed to native ETs (+ETs) or ETs opsonized with 166.67 nM anti-citH2A/H4 antibody or corresponding F(ab')2 fragments. Data are normalized to untreated controls (dotted line). One experiment was performed using a total of 5 donors, with a single measurement per donor. * *P*<0.05, ***P*<0.01, and ****P*<0.001. Statistical tests: Benjamini–Hochberg multiple comparisons test (A) and RM one-way ANOVA (B).

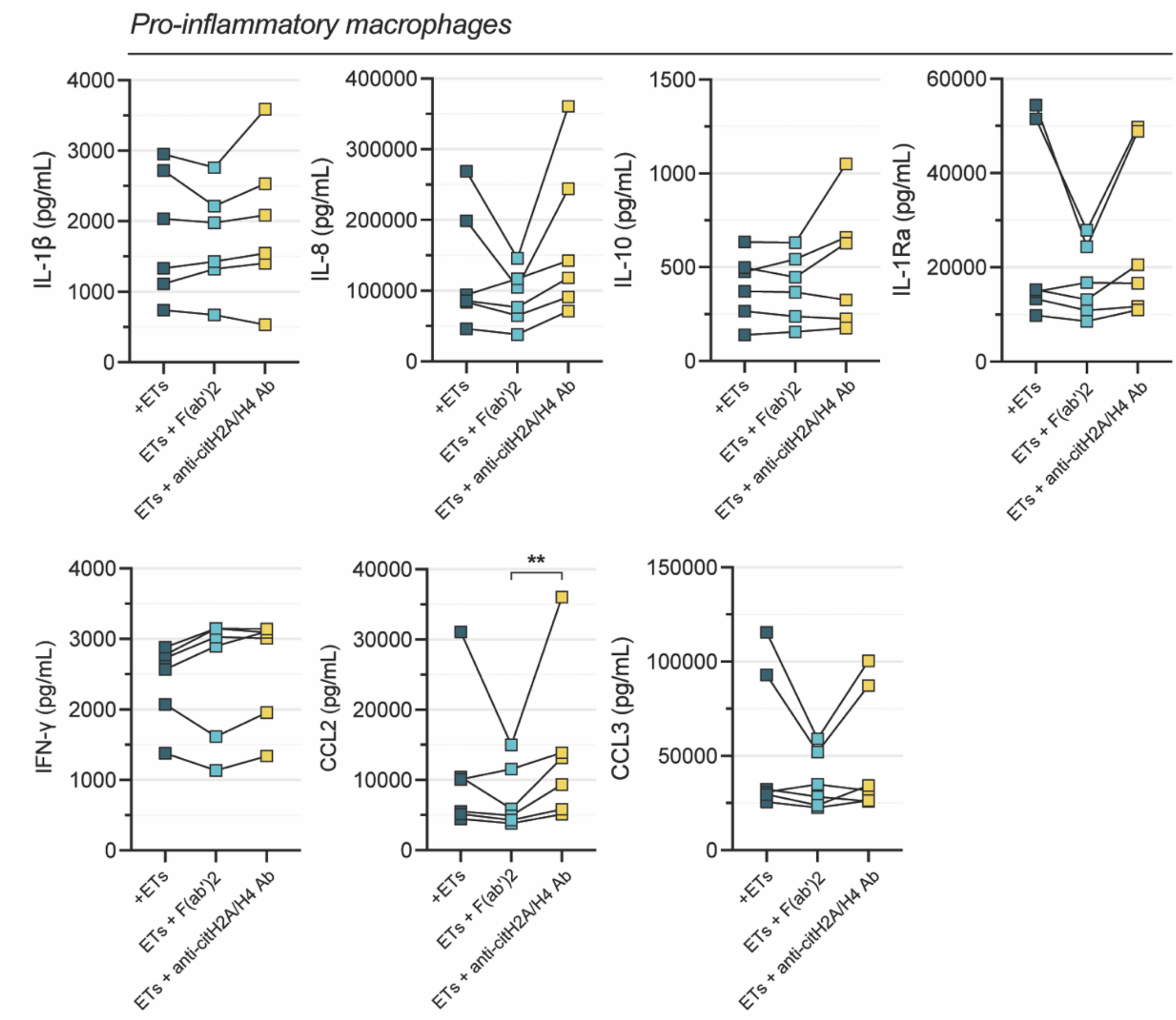

Supplementary Figure 7

IL-1β, IL-8, IL-10, IL-1Ra, IFN-γ, CCL2, and CCL3 levels in supernatant from pro-inflammatory macrophages exposed to native neutrophil-derived ETs (+ETs) or ETs opsonized with 166.67 nM anti-citH2A/H4 antibody or corresponding F(ab')2 fragments, following stimulation with LPS and IFN-γ. Two independent experiments were performed using a total of 6 donors, with pooled duplicates per donor.

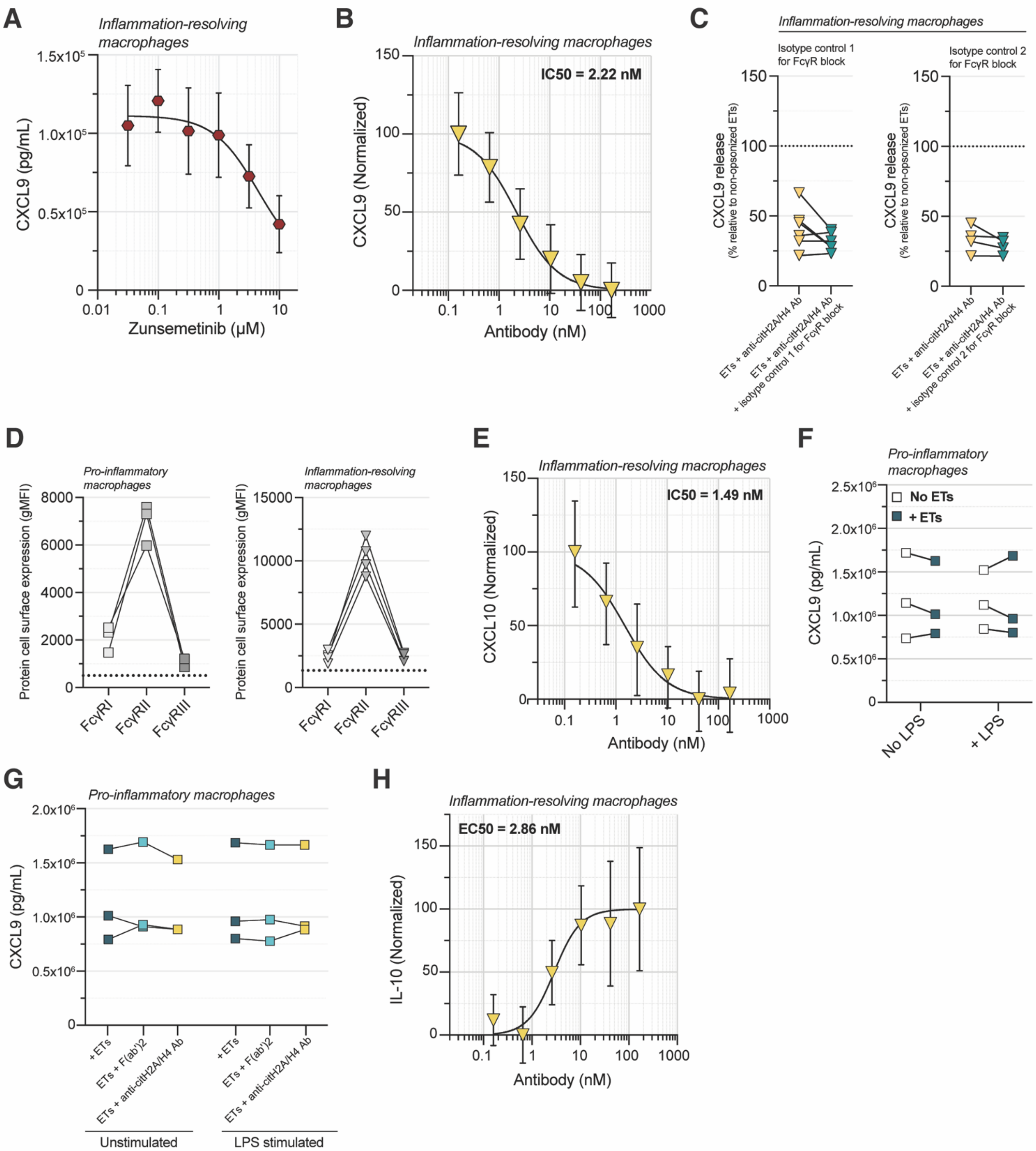

Supplementary Figure 8

Antibody-opsonized ETs attenuate *TLR-MAPK-dependent CXCL9 and CXCL10 release in macrophages*. **(A)** CXCL9 levels in supernatant from inflammation-resolving macrophages pretreated with indicated concentrations of a MAPK-activated protein kinase 2 inhibitor, followed by LPS stimulation. One experiment was performed using a total of 4 donors, with a single measurement per donor. **(B)** Normalized CXCL9 levels in the supernatant from inflammation-resolving macrophages pre-exposed to neutrophil-derived ETs opsonized with indicated concentrations of anti-citH2A/H4 antibody, followed by LPS stimulation. **(C)** Relative CXCL9 levels in the supernatant from inflammation-resolving macrophages pre-

exposed to ETs opsonized with 42.32 nM anti-citH2A/H4 antibody in the presence or absence of isotype control antibodies used for FcɣR-blocking experiments, followed by LPS stimulation. Data were normalized to CXCL9 levels released by macrophages pre-exposed to non-opsonized ETs, followed by LPS stimulation (dotted line). Two independent experiments performed using a total of 4-6 donors, with a single measurement per donor. **(D)** Quantification of FcɣRI, FcɣRII, and FcɣRIII surface expression on pro-inflammatory and inflammation-resolving macrophages. Dotted lines indicate background binding of the corresponding isotype control antibody. One experiment was performed using a total of 3-4 donors, with a single measurement per donor. gMFI, geometric mean fluorescence intensity. **(E)** Normalized CXCL10 levels in the supernatant from inflammation-resolving macrophages pre-exposed to ETs opsonized with indicated concentrations of anti-citH2A/H4 antibody, followed by LPS stimulation. Two independent experiments performed using a total of 4-6 donors, with a single measurement per donor. **(F)** CXCL9 levels in the supernatants from fully polarized (GM-CSF + LPS + IFN-γ) pro-inflammatory macrophages following LPS stimulation in the presence (+ETs) or absence of ETs (No ETs). One experiment was performed using a total of 4-6 donors, with a single measurement per donor. **(G)** CXCL9 levels in the supernatant from fully polarized pro-inflammatory macrophages pre-exposed to native ETs (+ETs) or opsonized ETs (166.67 nM) under unstimulated or LPS-stimulated conditions. One experiment was performed using a total of 3 donors, with a single measurement per donor. **(H)** Normalized IL-10 levels in the supernatant from inflammation-resolving macrophages pre-exposed to ETs opsonized with indicated concentrations of anti-citH2A/H4 antibody or corresponding F(ab')2 fragments. Two independent experiments were performed using a total of 4-6 donors, with technical duplicates per donor.
